# Heterologous calcium-dependent inactivation of Orai1 by neighboring TRPV1 channels modulates cell migration and wound healing

**DOI:** 10.1038/s42003-019-0338-1

**Published:** 2019-03-04

**Authors:** Carlos Ernesto Bastián-Eugenio, Arlette Bohórquez-Hernández, Jonathan Pacheco, Alicia Sampieri, Alexander Asanov, Jose Pablo Ocelotl-Oviedo, Adán Guerrero, Alberto Darszon, Luis Vaca

**Affiliations:** 1 Departamento de Biología Celular y del Desarrollo, Instituto de Fisiología Celular, Universidad Nacional Autónoma de México, Mexico City, 04510 Mexico; 2 TIRF Labs Inc., 106 Grendon Place, Cary, 27519 NC USA; 3 Departamento Genética del Desarrollo y Fisiología Molecular, Instituto de Biotecnología, Universidad Nacional Autónoma de México, Mexico City, 62250 Mexico; 4 Laboratorio Nacional de Microscopía Avanzada, Universidad Nacional Autónoma de México, Mexico City, 62250 Mexico

## Abstract

Store-operated calcium entry (SOCE) is an essential calcium influx mechanism in animal cells. One of the most important auto regulatory control systems involves calcium-dependent inactivation (CDI) of the Orai channel, which prevents excessive calcium influx. In the present study we analyze the role of two channels in the induction of CDI on Orai1. Here we show that calcium entering through freely diffusing TRPV1 channels induce strong CDI on Orai1 while calcium entering through P2X_4_ channel does not. TRPV1 can induce CDI on Orai1 because both channels were found in close proximity in the cell membrane. This was not observed with P2X_4_ channels. To our knowledge, this is the first study demonstrating that calcium arising from different channels may contribute to the modulation of Orai1 through CDI in freely diffusing single channels of living cells. Our results highlight the role of TRPV1-mediated CDI on Orai1 in cell migration and wound healing.

## Abstract (web summary)

Bastián-Eugenio et al. showed that calcium entering the cell via TRPV1, but not P2X_4_ channels, can induce calcium-dependent inactivation of Orai1. This inactivation impacts thrombin-induced cell migration and wound healing suggesting an important role of Orai1 modulation by TRPV1 channels.

## Introduction

The calcium ion (Ca^2+^) is a second messenger with a key role in numerous cellular processes^1^. Cells have developed many mechanisms to regulate this ion^2^. Store-operated calcium entry (SOCE) is the principal mechanism for calcium mobilization in non-excitable cells^3,4^. The prototypical store-operated calcium channel is the Ca^2+^ release-activated Ca^2+^ (CRAC) channel^5,6^. The essential components of CRAC are the endoplasmic reticulum (ER) Ca^2+^ sensor STIM1^7,8^ and the plasma membrane (PM) channel Orai^9^. In general, activation of inositol 1,4,5-triphosphate (IP_3_) receptors on the ER produces a rapid and transient release of Ca^2+^ from ER store. The resulting decrease of the Ca^2+^ concentration inside the ER is sensed by the EF-hand motif of STIM1, which then translocates to the PM, associating to Orai and inducing channel activation.

Orai activity is regulated through a negative feedback mechanism that maintains intracellular Ca^2+^ homeostasis and prevents excessive Ca^2+^ influx. Such a mechanism is known as Ca^2+^-dependent inactivation (CDI).

CDI consists of slow CDI (SCDI) and fast CDI (FCDI), which have different kinetics and sites of action. SCDI occurs gradually in tens of seconds after channel activation and has been reported to occur by global increases in cytosolic calcium concentrations^10^. The most important regulator of SCDI is the SOCE-associated regulatory factor (SARAF)^11^. Moreover, SCDI can be regulated by other factor such as caveolin, E-syt1, septin4, and PI(4,5)P_2_^12,13^.

FCDI take place within ~10–100 ms after channel activation and is controlled by Ca^2+^ binding to a site located ~8 nm from the channel pore^14,15^. FCDI is modulated by various factors, including the STIM1-Orai1 expression ratio^16^, an amino acid region negatively charged in STIM1 (residues 475–483)^17–19^, the intracellular loop II–III of Orai1^20^, the N-terminus of Orai1 (residues 68–91)^17,21^, and also probably the first 63 amino acids from Orai1^22^. Most interestingly, a single amino acid mutation alters FCDI in Orai1 channels rendering the channel CDI insensitive^21^.

To our knowledge, all the studies carried out to this date to understand and explore CDI have been conducted by artificially increasing intracellular Ca^2+^ via the patch clamp pipette or by measuring CDI with normal and reduced extracellular calcium concentrations, which reflects CDI induced by Ca^2+^ entering through the Orai channel pore (homologous CDI). Less studied are physiological sources of Ca^2+^, such as the contribution of other channels to CDI in Orai.

In the present study, we have explored other sources of Ca^2+^ arising from different channels that may play a role in Orai's CDI. We have found that Ca^2+^ entering the cell through TRPV1 channels induce strong CDI in Orai1, while Ca^2+^ entering through P2X_4_ purinergic channels does not. Super resolution studies indicate that Orai1 and TRPV1 are associated and move in close proximity to each other at the PM, while P2X_4_ and Orai1 do not. These results were confirmed by co-immunoprecipitation (CoIP) and Förster resonance energy transfer (FRET) studies between Orai1-TRPV1 and Orai1-P2X_4_.

All the results presented here strongly suggest that a close association between TRPV1 and Orai1 results in an elevated Ca^2+^ microenvironment near the Orai1 pore when TRPV1 channels are activated, which enhances CDI in Orai1. Because P2X_4_ and Orai1 are not found in close proximity at the PM, Ca^2+^ entering P2X_4_ channels do not induce CDI in Orai1, in spite the fact that Ca^2+^ entering through P2X_4_ channels contribute to increments in cytosolic Ca^2+^ concentrations.

These results have important physiological implications in the modulation of calcium influx in cells where TRPV1 and Orai1 channels coexist, such as astrocytes. We show that TRPV1 is an important modulator of Orai1 channel activity in cortical astrocytes by controlling CDI in this channel and thus reducing the amount of Ca^2+^ entering to the cell when TRPV1 and Orai1 are simultaneously or sequentially activated. This heterologous modulation of CDI plays a role in controlling cell migration and wound healing mediated by astrocytes.

## Results

### Engineering of Orai1-GCaMP3 to characterize CDI

In an attempt to identify how Orai1 may sense changes of Ca^2+^ in its vicinity, we engineered a fusion protein consisting of the green genetically encoded calcium indicator (GCaMP3) fused to the amino terminus of Orai1 channel (Orai1-GCaMP3). We use the red calcium indicator (R-GECO) to simultaneously measure changes in global calcium in the cytosol and compare it to local calcium changes reported by the fusion protein Orai1-GCaMP3, which would presumably report only calcium changes near the channel pore from Orai1.

**Fig. 1:**
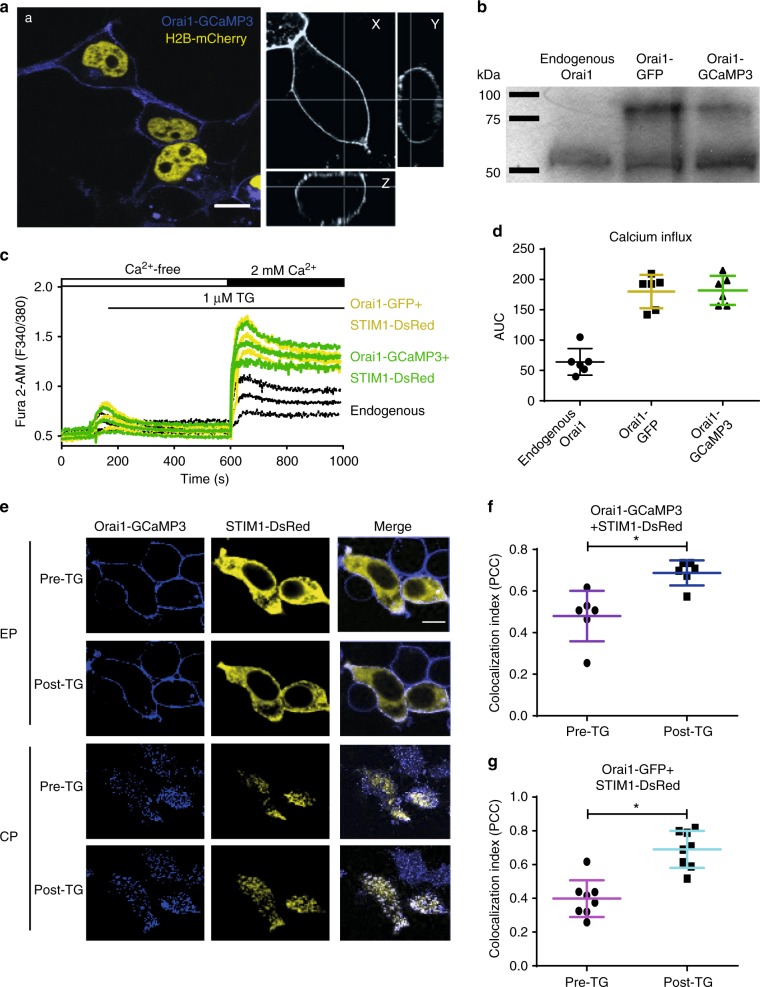
Developing and testing the Orai1-GCaMP3 sensor. **a** Representative confocal images of the fluorescence obtained with Orai1-GCaMP3 (blue) and histone 2B fused to mCherry as nuclear marker (yellow) expressed in HEK293 cells obtained from at least 3 independent transfections. Left panels show the 3D (*x*, *y*, and *z* axes) confocal projections from Orai1-GCaMP3 to illustrate its plasma membrane localization. Scale bar 5 µM. **b** Western blot analysis from HEK293 cells expressing the Orai1-GCaMP3 sensor and Orai1-GFP (representative blot from 3 independent blots). Notice the 55 kDa band corresponding to endogenous Orai1 and the 79 kDa band from Orai1-GFP and the Orai1-GCaMP3 sensor. **c** Cell population calcium measurements using a spectrofluorometer with HEK293 cells expressing Orai1-GFP + STIM1-DsRed (yellow) or Orai1-GCaMP3 + STIM1-DsRed (green) or measuring the endogenous SOCE (black). Measurements obtained from at least 5 independent transfections. Calcium increments induced by the application of thapsigargin (TG) in free extracellular calcium and SOCE measured after re-addition of 2 mM calcium to the extracellular solution. **d** Area under the curve (AUC) for fluorescence obtained after re-addition of 2 mM calcium (calcium influx) with endogenous Orai1 (gray), Orai1-GFP (yellow), and Orai1-GCaMP3 (green). **e** Representative confocal images of the puncta formation after TG application in cells expressing Orai1-GCaMP3 (blue) and STIM1-DsRed (yellow). Images shown before the application of thapsigargin (pre-TG) and after (post-TG) for the equatorial plane (middle) of the cell (EP) and the cortical plane (CP, the plane closest to the Petri dish bottom). Scale bar 5 µM. **f** Co-localization index (PC, Pearson’s correlation coefficient) for Orai1-GCaMP3 + STIM1-DsRed before and after TG. **g** Co-localization index (PCC) for Orai1-GFP + STIM1-DsRed before and after TG. In all cases, data shows the mean ± standard deviation from at least 6 independent transfections. Asterisks show *p* values of < 0.01. The statistical significance was *p* < 0.05. Statistics was performed by Student’s *t*-test

Firstly, we characterize Orai1-GCaMP3 to determine if the fusion protein behaves like a wild type Orai1 channel. Orai1-GCaMP3 is found primarily at the PM, similarly to Orai1 alone or Orai1-GFP used as controls (Fig. 1a). The expression of the fusion protein was identified also by western blot (Fig. 1b, for full-length blot see Supplementary Fig. 1). As expected, cells overexpressing Orai1-GCaMP3 or Orai1-GFP showed enhanced SOCE (Fig. 1c, d), indicating that the functionality of Orai1-GCaMP3 is indistinguishable from that of Orai1-GFP. This was clear when measuring the area under the curve (AUC) for calcium entry (Fig. 1d), but also with the peak increments in Fura-2 fluorescence from the same data (endogenous 0.43 ± 0.15, Orai1-GCaMP3 0.92 ± 0.18, and Orai1-GFP 0.99 ± 0.16, Fig. 1c).

Furthermore, Orai1-GCaMP3 forms regular puncta with STIM1 when internal calcium stores are depleted with thapsigargin (TG) (Fig. 1e–g). These results indicate that the function of Orai1 is unaltered by fusing the GCaMP3 calcium indicator.

**Fig. 2:**
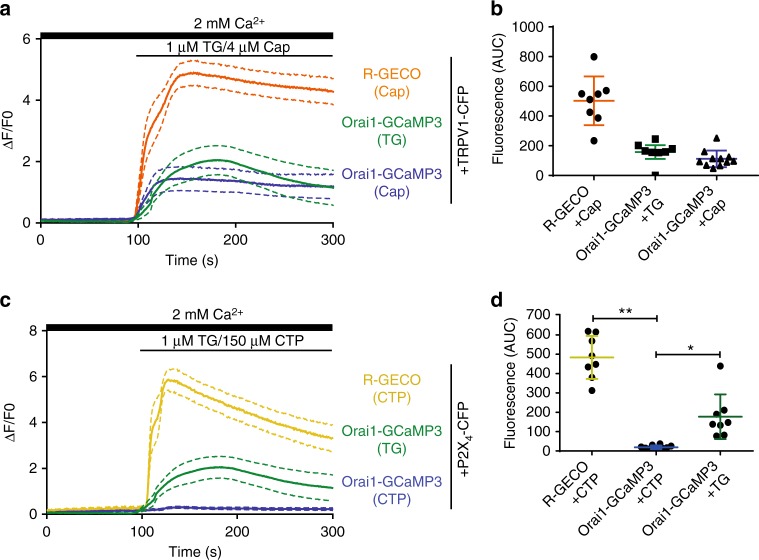
Orai1-GCaMP3 senses local calcium increments through TRPV1 but not through P2X_4_. **a** Calcium increments reported by R-GECO when the cells were exposed to capsaicin, Orai1-GCaMP3 with capsaicin and Orai1-GCaMP3 with thapsigargin (TG) from at least 12 independent transfections. **b** Area under the curve (AUC) for fluorescence of R-GECO with capsaicin (orange), Orai1-GCaMP3 with capsaicin (blue), and Orai1-GCaMP3 with TG (green). **c** Calcium increments reported by R-GECO when the cells were exposed to CTP, Orai1-GCaMP3 with CTP, and Orai1-GCaMP3 with thapsigargin (TG). **d** Area under the curve (AUC) for fluorescence of R-GECO with CTP (yellow, *n* = 8), Orai1-GCaMP3 with CTP (blue, *n* = 10), and Orai1-GCaMP3 with TG (green, *n* = 8). Notice that Orai1-GCaMP3 does not sense calcium increments with CTP while the R-GECO reports large calcium increments. In all cases, data shows the mean ± s.e.m. **p < 0.01, *p < 0.05. The statistical significance was *p* < 0.05. Statistics was performed by one-way ANOVA with Tukey’s post hoc test

We characterize further the calcium changes sensed by Orai1-GCaMP3 or by R-GECO present in the cytosol as a soluble protein. We proceeded to explore the ability of Orai1-GCaMP3 to sense calcium increments when other channels that permeate calcium are activated. We explored several channels and found that Orai1-GCaMP3 senses calcium arising from the activation of TRPV1 channel (Fig. 2a, b) but not from the P2X_4_ purinergic receptor (Fig. 2c, d). Most interestingly, a PM targeted calcium sensor (Lck-GCaMP5)^23^ detects calcium increments derived from the activation of both, TRPV1 and P2X_4_ channels (Supplementary Fig. 2). These results suggest that, even though both fusion proteins (Orai1-GCaMP3 and Lck-GCaMP5) are located at the PM, they may be contained within different microdomains conferring them differential sensitivity to calcium increments arising from different sources.

We decided to characterize in greater detail the interactions between Orai1-TRPV1 and Orai1-P2X_4_ in an attempt to identify the molecular mechanism responsible for the differential sensing of calcium by Orai1-GCaMP3 when TRPV1 or P2X_4_ are activated.

**Fig. 3:**
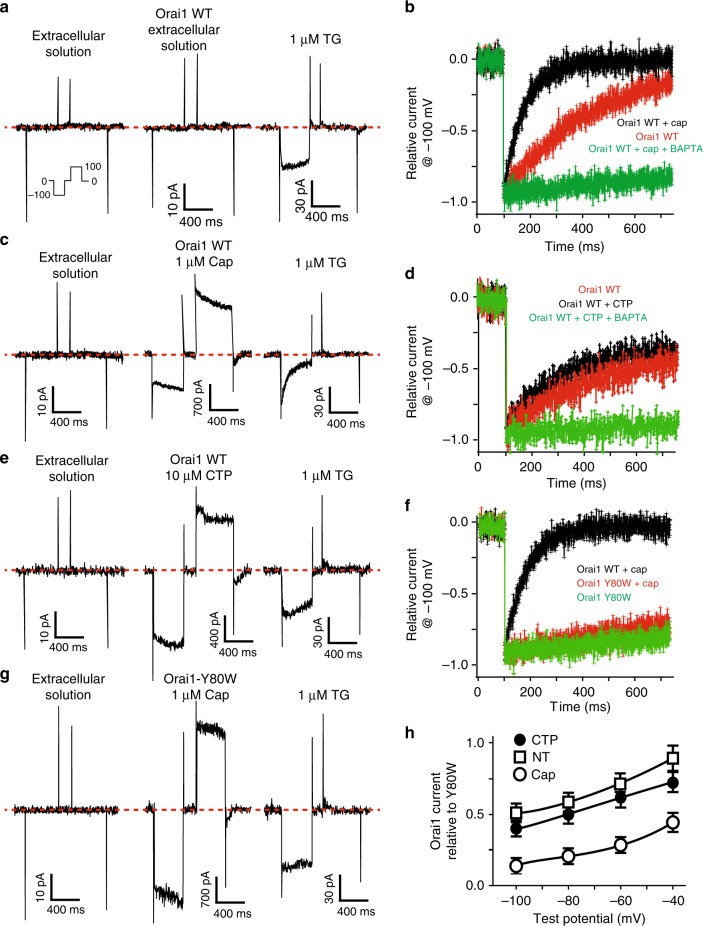
Activation of TRPV1 induces strong CDI in Orai1. **a** Perforated patch measurements in HEK293 cells expressing Orai1-GFP and STIM-DsRed. The sequential protocol consisted in the application of extracellular solution followed by a second control application of extracellular solution (ES) and finally thapsigargin. The second ES application will be replaced by either capsaicin or cytidine triphosphate (CTP) in the following experiments. In all cases, capsaicin or CTP were incubated for 3 min and then the cells were washed with fresh ES for 1–2 min prior to TG stimulation (TG was present for the remaining duration of the experiment). **b** Relative current illustrating the activation by applying a voltage step from 0 to −100 mV for cells expressing Orai1 and TRPV1 pre-stimulated with capsaicin (cap, black), Orai1 only with thapsigargin (red), and Orai1 and TRPV1 pre-stimulated with capsaicin in cells incubated with BAPTA-AM (green). **c** Perforated patch measurements using the sequential protocol pre-simulating with capsaicin in cells expressing Orai1-GFP and TRPV1-CFP. The middle panel shows the currents elicited by capsaicin stimulation (TRPV1). Panel to the right shows the subsequent activation of Orai1 with TG. **d** Relative current illustrating the activation of current by applying a voltage step from 0 to −100 mV for cells expressing Orai1 and activated by TG (red), cells expressing Orai1 and P2X_4_ and pre-stimulated with CTP (black) and cells expressing Orai1 and P2X_4_ and pre-stimulated with CTP and previously incubated with BAPTA-AM (green). Lines show mean ± standard deviation from at least 31 cells. **e** Perforated patch measurements using the sequential protocol in a cell expressing Orai1 and P2X_4_. **f** Relative current illustrating the activation for cells expressing Orai1 wild type (WT) with pre-stimulation with capsaicin (black) and cells expressing the CDI-resistant mutant Orai1 Y80W with (red) or without pre-stimulation (green). **g** Perforated patch measurements using the sequential protocol in cells expressing Orai1 Y80W and TRPV1. **h** Orai1 currents relative to mutant Orai1 Y80W at different voltages. Cells pre-stimulated with CTP (black circle), with capsaicin (open circle) or cells not exposed to capsaicin, only TG (NT). All experiments show the mean ± standard deviation from at least 20 cells

If Orai1-GCaMP3 is sensing increments in calcium upon activation of TRPV1 but not P2X_4_, then the calcium accumulating in the proximity from Orai1 should have an effect on Orai1 CDI. To explore this hypothesis, we developed a sequential activation patch clamp protocol to induce channel activity first on TRPV1 or P2X_4_ followed by the activation of Orai1 with TG and measure whole-cell currents in the perforated patch clamp mode. Control experiments consisting of perfusion with extracellular solution (Methods) provided a basal Orai1 activity and a control for CDI (Fig. 3a, b).

Activation of TRPV1 with capsaicin (Cap) followed by the activation of Orai1 with TG resulted in strong CDI (Fig. 3b, c). This phenomenon was not observed when we activated P2X_4_ with cytidine triphosphate (CTP) followed by the activation of Orai1 with TG (Fig. 3d, e). To further demonstrate that the rapid decay in Orai1 current was the result of CDI, we conducted experiments with the Orai1 mutant insensitive to CDI (Orai1-Y80W)^21^. Activation of TRPV1 followed by activation of Orai1-Y80W with TG did not induce significant CDI (Fig. 3f, g). Furthermore, using strong calcium buffering with BAPTA inside the cell (BAPTA-AM) further confirmed that the rapid reduction in Orai1 current was the result of CDI (Fig. 3b, d). The CDI observed in Orai1 after TRPV1 activation was evident at all voltages explored (Fig. 3h).

### Orai1 is in close proximity to TRPV1 but not to P2X_4_

**Fig. 4:**
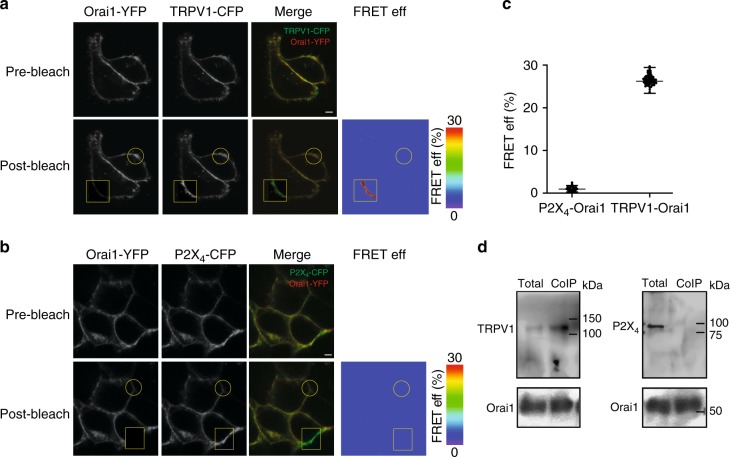
TRPV1 is in close proximity to Orai1. **a** Representative confocal images illustrating the expression of Orai1-YFP and TRPV1-CFP before (pre-bleach) or after (post-bleach) photobleaching of the acceptor (YFP). The square shows the area photobleached and the circle a control area not exposed to the photobleach procedure. We used both areas to calculate the FRET efficiency as indicated in Methods. Right panel shows the FRET efficiency (FRET eff) in pseudo color calculated from the acceptor photobleaching protocol (Methods). Scale bar 1 µM. **b** Representative confocal images illustrating the expression of Orai1-YFP and P2X_4_-CFP before (pre-bleach) or after (post-bleach) photobleaching of YFP. The square shows the area photobleached and the circle a control area not exposed to the photobleach procedure. FRET efficiency (FRET eff) in pseudo color calculated from the acceptor photobleaching protocol (Methods). Scale bar 1 µM. **c** FRET eff (%) obtained from at least 45 cells for P2X_4_-Orai1 and TRPV1-Orai1. Data show the mean ± standard deviation. **d** Co-immunoprecipitation analysis of cells expressing c-Myc-Orai1 and TRPV1-YFP or P2X_4_-CFP (for the entire blots check Supplementary Figures). Total indicates the protein that was not retained in the affinity column, thus reflects the protein that did not co-immunoprecipitate (Methods). Blots are representative examples from at least 3 independent blots

If calcium entering TRPV1 is inducing strong CDI in Orai1 but not calcium entering through P2X_4_, this result strongly suggests that TRPV1 and Orai1 must be in close proximity from each other. Indeed FRET analysis indicates that Orai1-YFP and TRPV1-CFP are within less than 10 nm from each other (Fig. 4a, c). FRET was not observed between Orai1-YFP and CFP-P2X_4_ (Fig. 4b, c). These results were confirmed by CoIP studies with Orai1-TRPV1 and Orai1-P2X_4_ (Fig. 4d, for full-length blots see Supplementary Fig. 3).

**Fig. 5:**
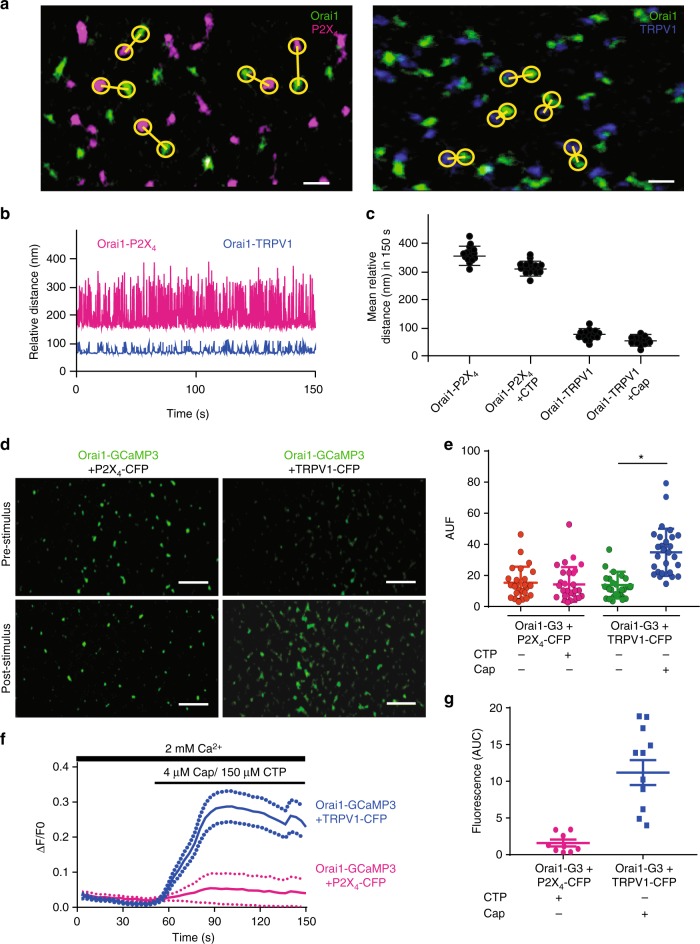
Super resolution studies reveal that TRPV1 and Orai1 move in close proximity in the plasma membrane. **a** Super resolution single channel TIRFM images from cells expressing Orai1 (green) and P2X_4_ (magenta) in the left panel or Orai1 (green) and TRPV1 (blue, right panel). Yellow circles show measurements between the nearest two channels for Orai1-P2X_4_ (left panel) or Orai1-TRPV1 (right panel). Scale bar 60 nm. **b** Relative distance over time for a single pair of channels measured in cells expressing Orai1-P2X_4_ (magenta) and Orai1-TRPV1 (blue). **c** Mean relative distance obtained from several hundred channels in cells expressing Orai1-P2X_4_ (magenta) with or without CTP and Orai1-TRPV1 (blue) with or without capsaicin. Notice that agonist stimulation (CTP or capsaicin) did not alter mean distances. **d** Super resolution single channel TIRFM images from cells expressing Orai1-GCaMP3 (green) + P2X_4_-CFP (left panels) or TRPV1-CFP (right panels) before and after stimulation with CTP or capsaicin. Notice the increment in the fluorescence of single Orai1-GCaMP3 channels after capsaicin stimulation but not with CTP. Scale bar 200 nm. **e** Fluorescence intensity in arbitrary units (AUF) reported by the Orai1-GCaMP3 sensor under the different stimulations. Notice that only cells expressing TRPV1 (T1-CFP) exposed to capsaicin produced an increment in fluorescence (blue bar). Capsaicin stimulation in cells without TRPV1 (orange and magenta bar) or cells expressing TRPV1-CFP but not exposed to capsaicin (green bar) did not result in changes in the fluorescence of Orai1-GCaMP3. **f** Fluorescence measurements of the entire field of view (containing dozens of individual channels) over time with cells expressing the Orai1-GCaMP3 sensor and TRPV1 with capsaicin (blue line) or cells expressing P2X_4_ and stimulated with CTP (magenta line). Lines show the mean ± standard deviations (dotted lines). Fluorescence from Orai1-GCaMP3 sensor increases only after capsaicin stimulation in cells expressing TRPV1. **g** Area under the curve (AUC) for the different conditions shown in panel **f**. Notice that only the fluorescence is observed in cells expressing the Orai1-GCaMP3 sensor + the TRPV1 channel and stimulated with capsaicin (green bar). *p < 0.01. The statistical significance was *p* < 0.05. Statistics was performed by Student’s *t*-test

To explore at the single channel level how calcium entering TRPV1 is sensed by our Orai1-GCaMP3 fusion protein, we conducted super resolution studies. Super resolution imaging showed individual Orai1, TRPV1, and P2X_4_ channels freely diffusing at the PM of living cells (Fig. 5a and Supplementary Movies 1 and 2). Measuring the distance between Orai1-TRPV1 and Orai1-P2X_4_ confirmed the results obtained by FRET and CoIP. The distance between individual Orai1 and TRPV1 channels was significantly smaller than that between Orai1-P2X_4_ (Fig. 5b). Average distances over time obtained from hundreds of single channels showed at least a 4-fold greater distance between Orai1 and P2X_4_ compared to Orai1 and TRPV1 (Fig. 5c). Furthermore, super resolution imaging of fluorescence increments using Orai1-GCaMP3 single channels showed that this fusion protein could sense calcium increments arising from TRPV1 but not P2X_4_ channels (Fig. 5d, e and Supplementary Movies 1 and 2). These differences in sensing calcium from TRPV1 or P2X_4_ were evident also when measuring the average change in fluorescence from hundreds of single Orai1-GCaMP3 channels (Fig. 5f, g).

### ANK1 of TRPV1 is essential for the association to Orai1 C terminus

**Fig. 6:**
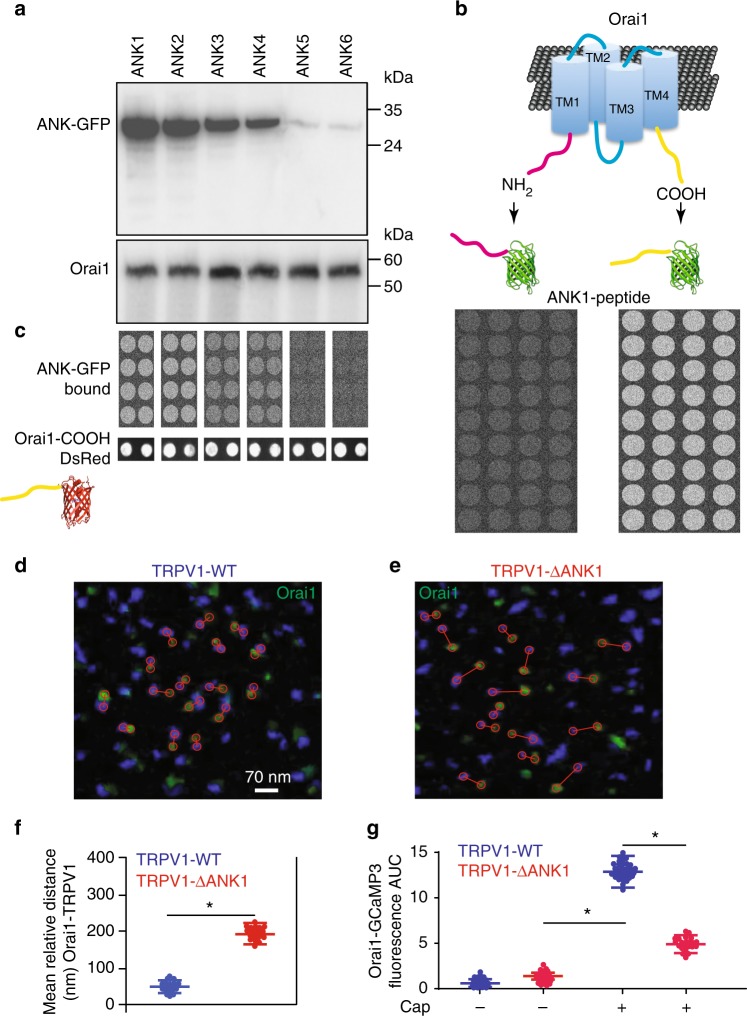
The first ankyrin domain from TRPV1 associates to the carboxyl domain from Orai1 to favor TRPV1-Orai1 association. **a** Co-immunoprecipitation analysis of cells expressing c-Myc-Orai1 and the 6 ankyrin domains fused to GFP (ANK1-ANK6). **b** Cartoon showing the fusion of the amino or the carboxyl terminus from Orai1 to GFP. Lower panels show the results of the peptide microarrays using ANK1 as bait. Notice that only COOH-GFP is captured by ANK1 but not NH2-GFP. **c** Peptide microarrays using the 6 ankyrin domains individually fused to GFP. In this case Orai1-COOH is used as bait (printed on the glass coverslip). Notice that Orai1-COOH retained ANK1-GFP, ANK2-GFP and less efficiently ANK3 and ANK4 but not ANK5 and ANK6. **d** Super resolution TIRFM images of cells expressing TRPV1 wild type (TRPV1-WT, blue) and Orai1 (green) or cells expressing TRPV1-ΔANK1 (right panel, **e**). Red circles show the distance between the closest pair of channels and the lines connect the pair. **f** Mean relative distance obtained from hundreds of individual channels for Orai1 with TRPV1-WT (blue) or TRPV1-ΔANK1 (red). Data shows the mean ± standard deviation. **g** Fluorescence increments reported by the Orai1-GCaMP3 sensor with and without capsaicin stimulation. Notice that the Orai1-GCaMP3 increases about half the fluorescence after capsaicin in cells expressing TRPV1-ΔANK1 (red bar) compared to the cells stimulated with capsaicin and expressing TRPV1-WT (green). This indicates that deletion of the first ANK domain results in Orai1 moving apart from TRPV1 and the Orai1-GCaMP3 sensor reporting less of the calcium that enters through the TRPV1 channel. *p < 0.01. The statistical significance was *p* < 0.05. Statistics was performed by one-way ANOVA with Tukey’s post hoc test

To identify the molecular structures responsible for the association between Orai1 and TRPV1 channels, we conducted a wide screening using peptide microarrays with a platform we have previously developed and tested^24^ (data available here^25^). Previous studies have highlighted the role of the ankyrin domains from TRPV1 in the association to regulatory proteins and scaffolds^26^. To evaluate the role of each of the 6 ankyrin domains from TRPV1 we produced fusion proteins of each domain to the green fluorescent protein (GFP) and conducted CoIP studies using Orai1 as bait. CoIP analysis indicated that the first 3 ankyrin domains associate to Orai1, although the first ankyrin domain (ANK1) showed to strongest interaction (Fig. 6a, for full-length blots see Supplementary Fig. 4). To explore further to what sequence from Orai1 was the ANK1 associating, we produced fusion proteins between the amino terminus from Orai1 and GFP (Orai1-NH2-GFP) and the carboxyl terminus from Orai1 (Orai1-COOH-GFP). Peptide microarray studies showed that the strongest interaction was between ANK1 and COOH-GFP (Fig. 6b). COOH-GFP interacts with the first 3 ankyrin domains from TRPV1, but the strongest interaction is with ANK1 (Fig. 6c). To ensure that the amount of Orai1-COOH deposited on each microarray spot was not affecting the binding of the different ankyrin domains, we produced a fusion protein of the carboxyl terminus from Orai1 fused to the red fluorescent protein (Orai1-COOH-DsRed). In this way, we can determine how much of the Orai1-COOH-DsRed was deposited on each microarray spot (using the red fluorescence channel) and the binding from the 6-ankyrin domains fused to GFP (using the green fluorescence channel), simultaneously (Methods). Below the strips of the microarray from the 6 ankyrin domains are illustrated examples of the Orai1-COOH-DsRed deposited on the microarray coverslip for each condition (Fig. 6c, lower panels labeled Orai1-COOH-DsRed)^25^.

To explore the role of the first 3 ankyrin domains in the entire TRPV1 protein, we produced individual deletions of these 3 ankyrin domains. Unfortunately, only the deletion of the first ankyrin domain produced channels that reached the PM, deleting beyond this first ankyrin domain resulted in channels that were retained in intracellular compartments. This finding was previously reported, showing that only the first amino acids from the amino terminus of TRPV1 can be deleted without affecting channel localization and function^26^. In particular, the ankyrin domain 3 plays a key role in channel tetramerization^27^. Nevertheless, we could explore the role of the first ankyrin domain on CDI using the deletion mutant TRPV1-ΔANK1, which is lacking the first ankyrin domain (Methods).

Super resolution imaging analysis showed that the relative distance between TRPV1-ΔANK1 and Orai1 was greater than that between TRPV1 wild type (TRPV1-WT) and Orai1 (Fig. 6d–f). Most interestingly, Orai1-GCaMP3 partially sense changes in calcium when TRPV1-ΔANK1 was activated with capsaicin compared to TRPV1-WT (Fig. 6g). Activation of TRPV1-WT and TRPV1-ΔANK1 with capsaicin results in robust increments in intracellular calcium reported by R-GECO produced as a soluble protein in the cytosol (Supplementary Fig. 5), discarding the possibility that the reduced sensing of calcium entering via TRPV1-ΔANK1 by Orai1-GCaMP3 may be the result of reduced calcium influx by the TRPV1-ΔANK1 deletion mutant. Furthermore, TRPV1-ΔANK1 does not co-immunoprecipitate with Orai1 (Supplementary Fig. 5).

Activation of TRPV1-ΔANK1 using our sequential activation patch clamp protocol showed only reduced CDI in Orai1 while TRPV1-WT produced strong CDI as previously shown (Fig. 3 and Supplementary Fig. 6). To discard any nonspecific effects of capsaicin on ionic currents, we conducted additional controls using the synthetic antagonist of capsaicin, capsazepine (Supplementary Fig. 5). Capsazepine stimulation had no effect on Orai1 CDI. Furthermore, the activation of TRPV1 with capsaicin in cells incubated with BAPTA-AM showed no effect on Orai1 CDI, strongly supporting the hypothesis that the CDI observed on Orai1 is the result of increments in intracellular calcium mediated by TRPV1 (Supplementary Fig. 5).

These results highlight the role of the first ankyrin domain in the association of TRPV1 to the carboxyl terminus from Orai1. This molecular interaction supports the formation of a complex between Orai1 and TRPV1, bringing the two channels in close proximity. This association between both channels favors the generation of a calcium microenvironment near the Orai1 channel pore when TRPV1 is activated, which modulates CDI on Orai1. The strong CDI observed in Orai1 channels induced by the activation of TRPV1 remains even after TRPV1 is not active anymore (Fig. 3c). These results suggest that calcium entering TRPV1 triggers the initial steps of CDI on Orai1 channels, but a sustained mechanism maintains CDI even when the influx of calcium through TRPV1 has been stopped (by washing out capsaicin). The sustain CDI can be explained by the fact that FCDI is modulated by Orai1-STIM1 interactions and not only high calcium near the Orai1 pore. Such Orai1-STIM1 interactions are mediated by the STIM1-Orai1 expression ratio^16^ an amino acid region negatively charged in STIM1 (residues 475–483)^17–21^ from Orai1^22^.

CDI may prevent excessive calcium entry into the cell when both channels (TRPV1 and Orai1) are activated simultaneously or sequentially.

To facilitate the study of the interactions between TRPV1 and Orai1 and the identification of the amino acid domains involved in this association, we conducted all the experiments described to this point in HEK293 cells. However, in order to assess the effect of TRPV1-induced CDI on Orai1 channels in a physiological model, we implemented a scratch-wound assay (SWA) using primary cultures of cortical astrocytes. We have previously shown that the majority of thrombin-activated calcium influx in cortical astrocytes is carried by Orai1 channels^28^. Cortical astrocytes express TRPV1 channels^29^. Furthermore, thrombin plays a key role in astrocyte migration and wound healing^30–32^.

**Fig. 7:**
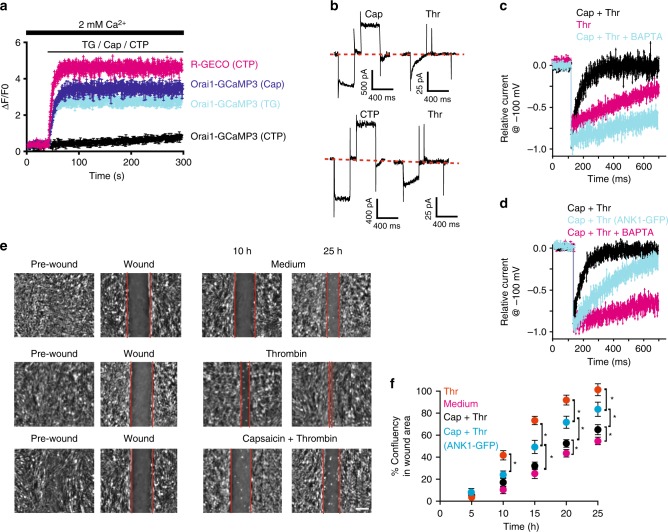
TRPV1 induced CDI on Orai1 modulates wound healing. **a** Cell population calcium measurements obtained with cortical astrocytes primary cultures transfected with Orai1-GCaMP3. Endogenous TRPV1 and P2X_4_ were used. Orai1-GCaMP3 senses calcium increments evoked with capsaicin (500 nM, blue) and TG (1 μM, cyan) but not with CTP (black). CTP induces robust calcium increments as reported by R-GECO (magenta). Data shows the mean ± standard deviation from at least 10 independent measurements obtained from 5 different cultures. **b** Stimulation with capsaicin (500 nM) prior to activation of Orai1 with thrombin induces strong CDI. CTP stimulation induces large P2X_4_ currents, however not CDI in Orai1 (activated by thrombin). **c** Perforated patch measurements of currents evoked by a voltage step from 0 to −100 mV in response to capsaicin followed by thrombin (black lines), thrombin alone (magenta) and capsaicin + thrombin in cells incubated with BAPTA-AM (cyan). BAPTA incubation strongly reduces CDI in cells stimulated with capsaicin and thrombin. Capsaicin incubated for 3 min and then the cells were washed with fresh PBS for 3 min prior to thrombin stimulation. **d** Perforated patch measurements of currents evoked by a voltage step from 0 to −100 mV in response to capsaicin + thrombin in cells expressing TRPV1 (black) and cells expressing also the ANK1-GFP domain (cyan) and cells incubated with BAPTA-AM (magenta). **e** Scratch-wound assay in cortical astrocytes. Representative photographs of the cell culture around the wound area are shown for 10 and 25 h after wound. Medium shows the cells that were maintained in DMEM. Thrombin indicates cells treated with thrombin (5 U mL^−1^ thrombin) for 3 min^42^. Cell cultures were exposed to capsaicin (500 nM) for 3 min and then incubated with thrombin (5 U mL^−1^ thrombin) for 3 min. **f** Percentage of confluency in wound area under the different treatments shown in **e**. Cells were also transfected with a construct carrying ANK1-GFP (blue). Data points show the mean ± standard deviation from at least 5 cell culture dishes for each condition. *p < 0.01. The statistical significance was *p* < 0.05 according to ANOVA

First, we validated that our Orai1-GCaMP3 sensor behaved in astrocytes similarly to what we have observed in HEK293 cells. Indeed, primary cultures of cortical astrocytes expressing our Orai1-GCaMP3 calcium sensor responded to capsaicin stimulation but not to the activation of P2X_4_ purinergic receptors with CTP (Fig. 7a). Nevertheless, astrocytes respond normally to CTP with a robust increment in intracellular calcium measured with the soluble calcium sensor R-GECO (Fig. 7a). Orai1-GCaMP3 senses also calcium increments induced with TG (Fig. 7a). All these results recapitulate what we observed with HEK293 cells expressing our Orai1-GCaMP3 construct (Fig. 2).

Capsaicin induces CDI on endogenous Orai1 currents from cortical astrocytes but not CTP stimulation (Fig. 7b). Furthermore, expression of the first ankyrin domain in astrocytes (ANK1-GFP) reduces the amount of CDI obtained with capsaicin pre-stimulation in our sequential perforated patch clamp protocol (Fig. 7c, d). Endogenous TRPV1 immunoprecipitate with endogenous Orai1 in cortical astrocytes (Supplementary Fig. 7), similarly to what we observed with the overexpression of these channels in HEK293 cells (Fig. 4d).

Using the SWA model, we have found that endogenous Orai1 participates in thrombin-induced cell migration and wound healing with cortical astrocytes, since transfection with a selective siRNA for Orai1^28^ prevents the effects of thrombin on wound healing (Supplementary Fig. 7).

Capsaicin stimulation previous to activation of Orai1 with thrombin significantly reduces wound healing (Fig. 7e, f), this effect is not observed with the synthetic antagonist of capsaicin, capsazepine (Supplementary Fig. 7). Expressing the ANK1-GFP domain in cortical astrocytes prevents the effect of capsaicin on thrombin-induced wound healing (Fig. 7e, f).

All these results strongly suggest that the activation of endogenous TRPV1 channels in cortical astrocytes modulate the activity of endogenous Orai1 channels by inducing CDI and reducing calcium entry through Orai1 channels. Enhanced CDI reduces thrombin-induced cell migration and wound healing. The results presented here provide the first evidence, to our knowledge, of the role of Orai1 in thrombin-activated cell migration and wound healing, while highlighting the role of CDI on Orai1 activity during this physiological process.

## Discussion

The present study identifies the molecular determinants of the association between TRPV1 and Orai1 channels, which favor heterologous CDI of Orai1 by calcium entering through single TRPV1 channels. Using super resolution studies on living cells, we observed that both channels move associated at the PM. This physical interaction results in relevant physiological modulation of channel activity (TRPV1 contributes to CDI on Orai1). This was not observed for another channel that does not associate to Orai1, the purinergic P2X_4_ receptor, even though activation of this receptor/channel results in robust increments in cytosolic calcium as reported by our cytosolic soluble sensor, R-GECO.

CDI is a regulatory process that prevents excessive calcium influx via Orai channels, which may have deleterious or toxic effects in the cell. Up until now, this phenomenon has been studied using electrophysiological whole-cell current measurements evoked by Orai channels. To induce CDI under these conditions, the extracellular and/or intracellular calcium concentrations have been artificially controlled. To our knowledge, this is the first report showing that a physiological source of calcium (e.g., activation of TRPV1) can induce CDI on Orai1 both in an heterologous expression system (HEK293 cells) and in cell that naturally express both channels (cortical astrocytes). We have shown that the induction of CDI is the result of the close proximity between Orai1 and TRPV1 at the PM, since the activation of a different channel (P2X_4_) did not induce CDI on Orai1. Furthermore, super resolution studies show that both channels (TRPV1 and Orai1) move in close proximity to each other. This was not observed for P2X_4_ and Orai1. The use of our local calcium reporter Orai1-GCaMP3 in super resolution studies shows that Orai1 single channels detect increments in calcium mediated by the activation of TRPV1 while freely diffusing at the PM. However, our Orai1-GCaMP3 sensor does not detect increments in calcium mediated after the activation of P2X_4_. These results obtained with super resolution studies indicate that Orai1 and TRPV1 are moving in close proximity to each other, maintaining a microenvironment where calcium entering via TRPV1 accumulates and induces CDI in Orai1.

Super resolution analysis showed at least a 4-fold greater distance between P2X_4_ and Orai1, when compared to the distance between TRPV1 and Orai1 (Fig. 5). The average distance between individual TRPV1 and Orai1 channels obtained from super resolution studies was around 50 nm. This distance contrasts to that observed with our FRET studies (less than 10 nm). The discrepancy can be explained by the resolution limits attained by both methods. While FRET detects distances between two proteins smaller than 10 nm (100 Å), the super resolution method (even though has gone beyond the light diffraction limit) it is still far away from the resolution of FRET. For example, STED (stimulated emission depletion) imaging has achieved a resolution of 20 nm when using organic dyes and 50–70 nm resolution when using fluorescent proteins^33^. The association of Orai1 and TRPV1 channels was evaluated also with CoIP studies. Even though all CoIP studies presented here are in agreement with the FRET and super resolution results, we would like to highlight the fact that the molecular weight obtained from Orai1 (≈55 kDa) differs from the predicted molecular weight for this protein (≈33 kDa). Thus, we would like to take the CoIP studies with caution, even though other reports have found similar discrepancies in the molecular weight for Orai1^34,35^. Feasible explanations for the higher molecular weight observed may involve glycosylation, phosphorylation, and other post-translational modifications of Orai1^36–38^.

We have identified the first ankyrin domain (ANK1) as a structure responsible for the association of TRPV1 to the carboxyl terminus from Orai1. Deletion of this ANK1 domain (TRPV1-ΔANK1) prevents TRPV1-mediated CDI on Orai1. Single Orai1-GCaMP3 channels do not longer sense calcium entering through TRPV1-ΔANK1.

A previous study shows that capsaicin inhibits Jurkat T-cell activation by blocking I_CRAC_^39^. Other studies have also shown that capsaicin or TRPV1 activation inhibits SOCE^40–43^. However, the molecular mechanism for these observations remained unknown until now.

CDI is a complex process, which involves the participation of several proteins such as SARAF^11^, caveolin, E-syt1, septin4, and PI(4,5)P_2_^13^. Furthermore, several amino acid regions have been identified in Orai1 and STIM1 to play a role in CDI^17–21^. This may be one of the reasons why CDI remained for several minutes after TRPV1 was not active anymore. Calcium entering through TRPV1 channel was the triggering event that initiated CDI in neighboring Orai1 channels, but other factors were involved in the sustained CDI even after the calcium source (TRPV1) was not active anymore (after washing out capsaicin).

In the present study, we focused our attention on the role of a different channel (TRPV1) in the induction of CDI on Orai1, to induced what we have named here heterologous CDI (as opposed to homologous CDI, which is induced by calcium entering via the Orai1 channel pore). We identified the ankyrin 1 domain from TRPV1 and the carboxyl terminus from Orai1 as sequences required for the association of these two channels. We cannot discard the participation of other proteins (as those mentioned above) or other amino acid regions in CDI. Further studies are required to evaluate the participation of other proteins and/or amino acid regions in Orai1 and TRPV1 in CDI.

Many cells and tissues express both TRPV1 and Orai1 channels, in particular cortical astrocytes. In these cells, activation of TRPV1 may modulate the subsequent (or concomitant) activation of Orai1 channels^44^. The role of astrocytes in chronic pain is well established^44^. We have previously shown that Orai1 mediates the thrombin response in cortical astrocytes^28^. Furthermore, thrombin induces cell migration and proliferation in astrocytes^30–32^.

In the present study, we have shown that the activation of endogenous TRPV1 channels with capsaicin induces CDI on endogenous Orai1 channels from cortical astrocytes. In this study, we showed that the heterologous modulation of Orai1 by TRPV1 via CDI plays a role in controlling injury-promoted astrocyte migration (Fig. 7). All these results provide the first evidence highlighting the role of heterologous CDI in controlling calcium entry via Orai1 and how this modulates migration in astrocytes primary cultures.

## Methods

### Plasmids and reagents

Capsaicin and the synthetic antagonist of capsaicin, capsazepine and cytidine triphosphate (CTP) were purchased from Sigma (St. Louis, MO). TG was purchased from Calbiochem (La Jolla, CA).

Plasmids Orai1-GFP, GCaMP3, STIM1-DsRed, H2B-mCherry, and Lck-GCaMP5 were purchased from Addgene (Cambridge, MA), R-GECO was a generous gift from Dr. Takeharu Nagai (Osaka University, Japan), TRPV1-CFP and TRPV1-YFP were kindly donated by Dr. Leon Islas-Suárez (Facultad de Medicina, UNAM), CFP-P2X_4_ was a generous gift of Dr. Jorge Arreola (Instituto de Fisica, UASLP). Full length complementary DNA of human-Orai1 was inserted into GCaMP3 between BamHI and EcoRI to generate Orai1-GCaMP3. All constructs were fully sequenced before using them.

### Ankyrin domains fused to GFP

Oligonucleotides were synthesized (Integrated DNA Technologies, Skokie, IL) to generate peptides corresponding to the amino acid sequences for the 6 ankyrin (ANK) domains from rat TRPV1 (Genbank NM_031982). Ankyrin domains were obtained from ref. ^45^.

Oligonucleotides were cloned in the pDrive plasmid after PCR amplification and cloned in frame at the 5′ of GFP. The resulting peptides were:

ANK1 110.152 (42 aa) RLYDRRSIFDAVAQSNCQELESLLPFLQRSKKRLTDSEFKDPE

ANK2 153.199 (46 aa) TGKTCLLKAMLNLHNGQNDTIALLLDVARKTDSLKQFVNASYTDSYY

ANK3 200.246 (46 aa) KGQTALHIAIERRNMTLVTLLVENGADVQAAANGDFFKKTKGRPGFY

ANK4 247.282 (35 aa) FGELPLSLAACTNQLAIVKFLLQNSWQPADISARDS

ANK5 283.331 (48 aa) VGNTVLHALVEVADNTVDNTKFVTSMYNEILILGAKLHPTLKLEEITNR

ANK6 332.358 (26 aa) KGLTPLALAASSGKIGVLAYILQREIH

Followed by the enhanced GFP sequence. All fusion genes were fully sequenced prior to use. For CoIP studies, the pcDNA 3.1 plasmid containing the nucleotide sequences for the 6 ankyrin domains individually fused to the GFP sequence (above) were transfected in HEK293 cells combined with the plasmid containing Orai1. For peptide microarray studies, the plasmids mentioned above were transfected in HEK293 cells maintained in suspension in 500 mL flasks to produce fusion proteins. Cells were harvested and sonicated to disrupt the cell membrane. Supernatants were immunoprecipitated using the commercial anti-GFP antibody (632376, Clontech, Mountain View, CA). Aliquots of the 6 ankyrin domains fused to GFP were utilized for TIRFM peptide microarray studies as previously described^46^. Briefly, silica glass was coated with a 0.01 mg mL^−1^ poly-D-lysine (Sigma, St. Louis, MO). Orai1-dsRED-COOH peptide was spotted on the microarray slide using a peptide solution of 500 ng in 50 μL. Approximately 2 μL were manually deposited on each spot using a pipette. Spots were allowed to dry for 20 min at room temperature prior to starting the TIRFM microarray experiment. Microarray slides were mounted on the lg-TIRFM closed chamber (TIRF Labs, Cary, NC).

A similar PCR strategy was conducted to produce the fusion proteins between the amino terminus from Orai1 and GFP (NH_2_-GFP), the carboxyl terminus from Orai1 (COOH-GFP), and the carboxyl terminus from Orai1 fused to the red fluorescent protein (DsRed, Takara Bio, USA). Production and purification of recombinant fused proteins followed the procedure described above. The Orai1-COOH domain contained the last 38 amino acids (amino acid 200–238) with the sequence: HKTDRQFQELNELAEFARLQDQLDHRGDHPLTPGSHYA (Genbank: AAH13386.1). Followed by the GFP or dsRED protein (Takara Bio, USA). The amino terminus of Orai1 contained the first 54 amino acids (1–54, Genbank: AAH13386.1): MSLNEHSMQALSWRKLYLSRAKLKASSRTSALLSGFAMVAMVEVQLDADHDYPP. Followed by the GFP protein. The deletion mutant TRPV1-RPV1-was produced by PCR amplification of TRPV1 cDNA using a forward oligonucleotide that started after nucleotide 536 (amino acid 153).

### Cell culture and transfection

Human embryonic kidney 293 cells (HEK293; ATCC) were cultured using Dulbecco's modified Eagle's medium (DMEM) (GIBCO) supplemented with 10% (V/V) fetal bovine serum, 50 µg mL^−1^ penicillin–streptomycin and maintained at 37 °C in a humidified atmosphere with 5% CO_2_.

Transient transfection was performed using Lipofectamine 2000 (Invitrogen) according to manufacturer instruction using cells seeded to 80% confluence in Optimem medium (GIBCO). Before imaging, culture medium was changed in Krebs solution (119 mM NaCl, 2.5 mM KCl, 1 mM NaH_2_PO_4_, 1.3 mM MgCl_2_, 20 mM HEPES, 11 mM glucose, 1.8 mM CaCl_2_, 500 µM EGTA and adjusted to 7.4 pH).

### Western blot

Proteins of cells transfected with Orai1-GCaMP3 or Orai1-GFP were separated using SDS-Page with 10% acrylamide and transferred to the membrane using 110 V for 60 min in a wet chamber. A specific antibody was used to identify Orai1 (ab59330, Abcam) and for TRPV1 the antibody used was VR1 (sc-398417) from Santa Cruz Biotechnology. Primary antibody was incubated with agitation at 4 C° for 1 h. The secondary antibody was incubated for 2 h with agitation at room temperature. Signal was acquired using X-ray films and then the image was digitalized and analyzed using ImageJ software. Full-length blots are included in Supplementary Figures.

### Confocal microscopy

HEK293 cells transfected with the different constructs and channels were plated on 25 mm coverslips. Cells were imaged in Krebs–Ringer’s solution. TG (1 μM; Calbiochem) was used for store depletion. Image acquisition for co-localization experiments was conducted 12 min after store depletion. Enhanced GFP (EGFP), GCaMP3, DsRed, and R-GECO were excited at 488, 488, 494, and 594 nm, respectively. Fluorescence emission was collected at 500–540 nm for EGFP and GCaMP3 and 605–650 nm for DsRed and R-GECO. All images were captured at room temperature with a ×60, 1.40 numerical aperture oil-immersion objective lens controlled by Fluoview Olympus software on a FV10i confocal microscope (Olympus imaging). Pearson's correlation coefficient was calculated with Fluoview Olympus software.

### Ca^2+^ measurements

Calcium imaging was performed in 25 mm coverslip with cells transfected with the plasmid of choice indicated in the figures. Cells expressing Orai1-GCaMP3 in Krebs–Ringer solution with Ca^2+^ were exposed to TG (1 µM) to induce store depletion. Capsaicin (4 µM) was utilized to activate TRPV1 channels and 150 µM cytidine triphosphate (CTP) to open P2X_4_ channels. Calcium dynamics were measured in individual cells (at least 20 per coverslip per condition) using a wide-field inverted IX81 Olympus® microscope with a ×60 1.42 NA oil immersion objective, MT-20 illumination system, 420/10, 484/25 excitation filter, and 450, 550/25, 520 nm/40 bandpass and 605/40 bandpass emission filter with an EMCCD camera iXon-897 (Andor Technology, South Windsor, CT, USA). Sampling was acquired each second during 300 s. The acquired images were analyzed using Olympus Cell-R software. To measure calcium increments in cell populations, we loaded HEK293 cells with FURA-2 AM (Molecular Probes) at 2 μM final concentration. FURA-2 AM was incubated for 30 min at 25 °C. Calcium-free Krebs solution contained (119 mM NaCl, 2.5 mM KCl, 1 mM NaH_2_PO_4_, 1.3 mM MgCl_2_, 20 mM HEPES, 11 mM glucose, 1 mM EGTA and adjusted to 7.4 pH). Then cells were rinsed with the same solution and were exposed to 1 µM TG to induce store depletion and Orai1 activation. Ca^2+^ entry was measured through an Aminco-Bowman luminescence spectrometer (Thermo Electron, Madison, WI). Dual excitation wavelength was selected at 340 and 380 nm and emission was collected at 510 nm. Fluorescence sampling was acquired each 10 s during 1000 s.

### FRET measurements

The cells were seeded on 25 mm coverslips and co-transfected with TRPV1-CFP and Orai1-YFP. We performed FRET measurements using the acceptor photobleaching protocol with TRPV1-CFP as donor and Orai1-YFP as acceptor, as previously described^4^.

For acceptor photobleaching, the images were acquired with an inverted Olympus FV1000 confocal microscope. The photobleaching of the acceptor was performed exposing the selected ROI to the laser at 50% of its maximum intensity (ROI indicated by a yellow square in Fig. 4), at a wavelength of 492 nm. This wavelength does not affect the donor as the excitation of GFP beyond 480 nm is negligible. The photobleaching protocol consisted of continuous excitation at 492 nm for 4 min, during this time, the fluorescence emission of CFP and YFP were monitored using the confocal microscope. Photobleaching excitation was alternated with image acquisition of CFP (460 nm) and YFP (520 nm). We used a 1.45 NA ×100 objective (Olympus, Japan) for image acquisition.

FRET efficiency, which indicates the percentage of excitation photons that contribute to FRET, was calculated measuring the fluorescence intensity in a ROI, before and after photobleaching the acceptor (Orai1-YFP), we also measured the fluorescence of acceptor and donor in a different ROI (without the photobleaching protocol applied, indicated by a yellow circle in Fig. 4) to correct for photobleaching that might occur during acquisition unrelated to the photobleaching protocol. FRET efficiency was calculated with the formula *E*_FRET_ = (*D*_post_ − *D*_pre_)/*D*_post_, where *D*_pre_ and *D*_post_ are YFP fluorescence before and after the photobleaching protocol, respectively. The *E*_FRET_ calculated from the area not subjected to photobleaching (indicated by a yellow circle in Fig. 4) was considered the baseline. This baseline value was indistinguishable from the *E*_FRET_ obtained between Orai1-YFP and CFP-P2X_4_ (Fig. 4). For a detailed description of the protocol, please refer to ref. ^47^.

### Electrophysiology

HEK293 cells expressing the different constructs described in Fig. 3 were placed on coverslips coated with poly-lysine (Sigma). Cells were studied between 24 and 48 h post-transfection. Coverslips were mounted on an open perfusion chamber (TIRF Labs, Cary, NC). The patch clamp amplifier used for whole-cell recordings was the EPC-10 (Heka Electroniks, Germany). The patch clamp pipettes were prepared from Corning 7052 glass and had a resistance of 1–5 MΩ when filled with the pipette solution (see below). An Ag/AgCl electrode was utilized to attain electrical continuity and was connected to the bath solution via a KCl-agar bridge. TG was applied using a multibarrel perfusion system driven by gravity (TIRF Labs, Cary, NC).

Whole-cell currents were studied in the perforated patch mode. Amphotericin B was dissolved in 50 μL DMSO to give a 60 mg mL^−1^ stock solution and stored at −20 °C until use. The amphotericin B stock solution was dissolved to a final concentration of 0.24 mg mL^−1^ in a syringe containing the pipette solution: cesium aspartate 120 mM, EGTA 5 mM, HEPES 10 mM, MgCl_2_ 2 mM, and NaCl 8 mM; pH to 7.2 adjusted with CsOH. The bath solution contained NaCl 120 mM, tetraethylammonium chloride (TEA-Cl) 10 mM, CaCl_2_ 10 mM, MgCl_2_ 2 mM, glucose 30 mM, and HEPES 10 mM; pH to 7.2 adjusted with NaOH. Osmolarity of both solutions was adjusted to 310 mOsm with mannitol (Sigma).

To determine Orai1 current inactivation, the cell was first stimulated with either 1 μM capsaicin (Cap) or 10 μM cytidine triphosphate (CTP) for approximately 1 min. The solution was removed and washout for 1–2 min with bathing solution before stimulation with 1 μM TG. Relative inactivation was obtained after obtaining the current density relative to the non-inactivating Orai1 Y80W mutant.

Current density was obtained after dividing the current from each cell by the cell capacitance (measured directly from the amplifier readout). The amplifier provided current density in real time calculated via the Patchmaster software and EPC10 electronics (Heka Electroniks, Germany). For time courses studying Orai1 whole-cell current activation, TG was applied while the cell membrane potential was held at −100 mV. All whole-cell current illustrated in Fig. 3 represents the mean ± standard deviation (SD) from at least 30 independent cells obtained from 4 different days and transfections.

Orai1 whole-cell currents were imported into Igor pro v. 7 (Wavemetrics, Oregon) for further analysis and plotting. Final figures were created with Adobe Illustrator (Adobe Systems).

### Super-resolution microscopy

The total internal reflection fluorescence (TIRF) excitation (cellTIRF Illuminator; Olympus) system was mounted on an Olympus IX81 inverted microscope for all super resolution experiments reported here. The excitation angle was set up to attain the critical angle for total internal reflection, which provided a penetration of less than 100 nm. Cells were continuously illuminated using excitation sources depending on the fluorophore studied. Blue (CFP-P2X_4_) and green (Orai1-YFP) were excited with either a 405 and 491-nm diode-pumped solid-state laser, respectively. Cells were continuously illuminated using the following excitation sources: blue (CFP-P2X_4_) and green (Orai1-YFP) were excited with either a 405 and 491-nm diode-pumped solid-state laser, respectively. The maximum laser power, measured at the back of the focal plane of the objective lens, ranged between 20 and 25 mW, depending on the laser line used. Excitation wavelength and intensity were modulated using the Xcellence software v.1.2. A multiband laser cube set was used to discriminate the selected light sources (LF 405/488/561/635 A-OMF, Bright Line; Semrock). The microscope was equipped with an Olympus UApo N ×100/1.49-numerical-aperture oil-immersion objective lens with an extra ×1.6 intermediate magnification lens. All movies were recorded on a crop chip mode (512 × 32 pixels) of an EMCCD camera (Andox, Ixon 888) at 100 nm per pixel.

For each independent experiment, sub-diffraction images were derived from the Super Resolution Radial Fluctuation (SRRF) analysis^48^. Reconstructions contained thousands of TIRF images acquired within the evanescent field. All images were collected using stroboscopic illumination, alternating between laser lines in a per-frame basis, each with an exposure time of 1–5 ms.

Each serial stack (300 images block) was analyzed using the NanoJ-core and NanoJ-SRRF plugins of Image J^48,49^. For the analysis the following parameters were utilized: ring radius 0.5, radiality magnification 5, axes in ring 10; all other parameters were set up as the default options in NanoJ-core and NanoJ-SRRF plugins. The radiality maps were drift corrected using pre-calculated drift tables obtained with the Estimate Drift tool of NanoJ-SRRF, considering an average of 300 images. These drift-corrected radiality maps were then finally integrated on a super-resolution image by calculating the Temporal Radiality Pairwise Product Mean.

To obtain distances between Orai1 and TRPV1 or Orai1 and P2X_4_ overtime, we used the tracking image correlation method^50^. For this purpose, HEK293 cells were transfected with a combination of Orai1-YFP and TRPV1-CFP or Orai1-YFP and CFP-P2X_4_, respectively. The signal of individual fluorophores was tracked overtime by alternating the excitation wavelengths, as described above. Temporal blocks of 300 images were analyzed. Tracking image correlation (TrIC) computes a colocalization analysis on single particles along their trajectory based on local image cross-correlation. The accuracy of the correlation is limited to the SRRF, which in our case is between 40 and 60 nm. All of the analysis was programmed with MATLAB (The MathWorks, Natick, MA) using the original algorithms described by Dupont and collaborators^50^. Unlike the original study, which tracked particles in the 3D space, we conducted only 2D tracking within the depth of the evanescent wave (less than 100 nm). The depth resolution of TIRFM (less than 100 nm) provided high accuracy when determining the 2D spatial localization of the particles when compared to confocal microscopy, which in the best case scenario has a depth resolution of around 600 nm^51^.

### TIRFM microarrays

For peptide microarrays studies using ankyrin domains fused to GFP, we utilized a novel system specifically developed for this purpose based on total internal reflection microscopy (TIRFM)^46^. We have documented extensively this method elsewhere^46^. Briefly, purified peptides (either the ANK1 domain (see above) or the Orai1-COOH-DsRed terminal domain) were printed using a micropipette on conventional glass microarray slides previously coated with a film of 0.01 mg mL^−1^ poly-D-lysine (Sigma, St. Louis, MO). The microarray-closed chamber was bathed with a solution containing the ANK domains fused to GFP (see above) at a final concentration of 1 μM. After 3 min incubation, the chamber was bathed with saline solution and fluorescence signal was collected for 1 min. Excitation was in TIRFM mode using the LG-TIRFM system (TIRF Labs, Cary, NC). Excitation was alternated between 405 nm (GFP excitation) and 594 nm (DsRed excitation). DsRed emission was used to evaluate the amount of Orai1-COOH-dsRED deposited on each microarray spot. Microarray spots containing less than 80% of the average DsRed fluorescence were discarded because insufficient Orai1-COOH-dsRED was deposited in those spots. Emission of GFP (D505/40 m) and DsRed (ET620/60 m) were collected using high-quality single band emission filter from Chroma (Chroma Technology Corporation, Bellows Falls, VT, USA). For details on our TIRFM microarray system and protocols for micro-perfusion and fluorescence detection, please refer to ref. ^24^.

### Co-immunoprecipitation

Plasmids c-Myc-Orai1 and TRPV1-YFP or CFP-P2X_4_ were overexpressed in HEK293 cells. Cells were washed twice with PBS, then the cells were lysed with TNI lysis buffer^52^ (0.5% Igepal CA-630, 50 mM Tris pH 7.5, 250 mM NaCl, 1 mM EDTA, 1× Complete ULTRA-EDTA-free protease Inhibitor cocktail) during 30 min at 4 °C with agitation and sonicated using a bath sonicator (55 kHz, 5 min). The samples were centrifuged 40 min (4 °C, 18,000×*g*) and the supernatant was incubated overnight with sepharose resin beads coupled to anti-c-myc antibody. Proteins were recovered by centrifuging the beads (4 °C, 500×*g*). Bound proteins were eluted with elution buffer^52^ (0.2 M glycine pH 2.3 and 0.5% Igepal CA-630) during 30 min at 37 °C. The proteins were analyzed by western blot using specific antibodies c-myc (Invitrogen, MA1-21316) for c-Myc-Orai1 (50 kDa), VR1 (Santa Cruz, 398417) for TRPV1-YFP (125 kDa) and P2X_4_ (Abcam, 134559) for CFP-P2X_4_ (71 kDa), all the antibodies were used according to the manufacturer’s recommendations. For endogenous Orai1 and TRPV1 from astrocytes we use the following protocol. Cells from 5 to 7 100 mm Petri dishes with confluent monolayers of primary culture astrocytes were rinsed and cells were detached mechanically. All cells from the Petri dishes were concentrated and lysed using TNI lysis buffer during 30 min with agitation at 4 °C and sonicated using a bath sonicator (55 kHz, 5 min). The samples were centrifuged 40 min (4 °C, 18,000×*g*) and the supernatant was incubated overnight with sepharose resin beads coupled to anti-Orai1 antibody. Proteins were recovered by centrifuging the beads (4 °C, 500×*g*). Bound proteins were eluted with elution buffer during 30 min at 37 °C. The proteins were analyzed by western blot using the specific antibodies described above.

### Scratch-wound assay

Primary cortical astrocytes were isolated from rat brains^28^. All procedures for maintaining the rats and for isolation of astrocytes were approved by the Animal Care Committee of the Instituto de Fisiología Celular, Universidad Nacional Autónoma de México. Animal care was performed according to the International Guiding Principles for Biomedical Research Involving Animals (Council for International Organizations of Medical Sciences, 2010). Cortical astrocytes cultures were obtained from 1-day-old Wistar rat pups provided by our animal facility center. The astrocytes were isolated from the brain cortex of 6–8 pups. Brains were placed in Krebs solution and cut in small slices. The tissue was dissociated with a solution containing trypsin (4800 U mL^−1^, Sigma, St. Louis, MO) for 10 min. Cells were mechanically dispersed by passage through 80 μm nylon mesh and resuspended in Eagle’s medium (Gibco, Grand Island, NY) supplemented with 10% heat-inactivated fetal bovine serum (Gibco), 2 mM glutamine (Sigma), 50 U mL^−1^ penicillin (Gibco), and 50 μg mL^−1^ streptomycin (Gibco). The dissociated astrocytes were plated on 35 mm plastic Petri dishes and incubated at 37 °C in humidified 5% CO_2_, 95% air atmosphere for 2 weeks.

When indicated astrocytes were transfected with the constructs described in the figure legends^28^. For RNA interference experiments, astrocytes cultures of 80% confluence in 35 mm plates were transfected with a set of three oligonucleotides of Stealth siRNAs for Orai1 (RSS357633, RSS357634, RSS357635) purchased from Invitrogen. A Stealth RNAi scramble negative control (Invitrogen) was used in parallel experiments. siRNAs transfection was carried out on primary astrocytes using Lipofectamine 2000 and Opti-MEM reduced serum medium according to the manufacturer’s instructions (Invitrogen).

For the SWA, we followed the next protocol. Cover glass chambers (ThermoScientific, Rochester, NY) were coated with 0.01 mg mL^−1^ poly-D-lysine (Sigma, St. Louis, MO). Confluent primary cortical astrocytes were seeded onto chambers^28^. Once confluent, astrocytes were serum-deprived overnight. A single scratch with a 1 mL pipet tip was made through the astrocyte monolayer, cells were rinsed three times with serum-free media. The scratch area was marked with a sharpie for later identification and positioning in the microscope. Images of cells were obtained with a low magnification ×10 objective attached to an inverted IX81 microscope (Olympus). A low-resolution 5 MP USB c-mount microscope camera (Zowaysoon) attached to the microscope c-port was used to acquire individual images of the scratch wound area at 0 time (pre-wound), immediately after the scratch wound, 5, 10, 15, 20, and 25 h after scratch wound. After every image acquisition cells were placed in the CO_2_ incubator.

### Reporting summary

Further information on experimental design is available in the Nature Research Reporting Summary linked to this article.

## Supplementary information

Supplementary Information

Description of Additional Supplementary Files

Supplementary Movie 1

Supplementary Movie 2

Reporting Summary

## Data Availability

All constructs used in the present study are available upon request. Super resolution image files are too big (each file is over 1 terabyte in size) to be included in any archiving system but are available upon request. Microarray data is publicly available (10.6084/m9.figshare.7621955.v1). Other data that support the findings of this study are available from the corresponding author upon reasonable request.
