# Supplementary material for: Heterologous calcium-dependent inactivation of Orai1 by neighboring TRPV1 channels modulates cell migration and wound healing
Source: Commun Biol. 2019 Mar 4;2:88. doi: 10.1038/s42003-019-0338-1 (PMC6399350; doi:10.1038/s42003-019-0338-1)
Supplement: Supplementary file 1 — Supplementary Information [file 42003_2019_338_MOESM1_ESM.pdf]

Supplementary Figure 1

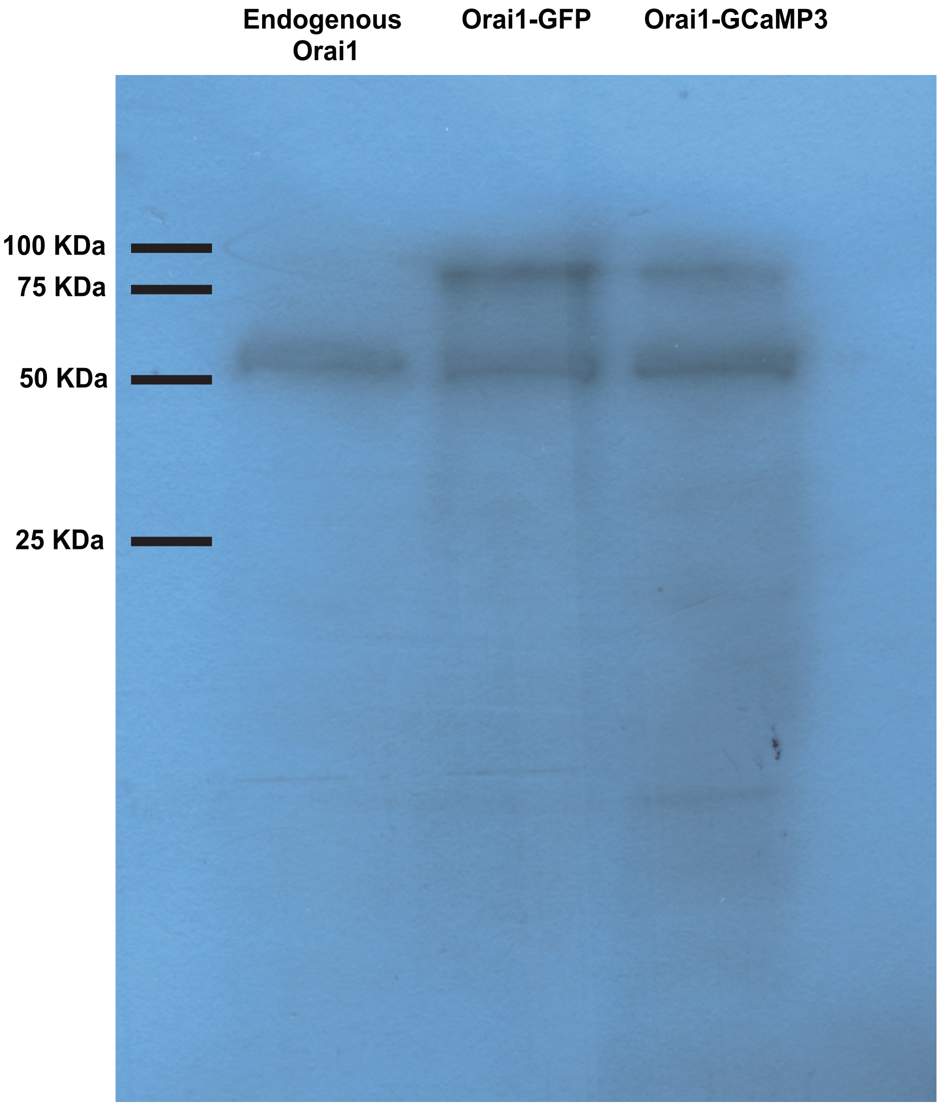

Supplementary Figure 1. Full length blots from Orai1, Orai1-GFP and Orai1-GCaMP3. Representative full-length blot showing the bands recognized by a specific Orai1 antibody (ab59330, Abcam). Line 1 (left) untransfected HEK293 cells showing endogenous Orai1. Line 2 (middle) HEK293 cells expressing Orai1-GFP. Line 3 (right) HEK293 cells expressing Orai1-GCaMP3.

Supplementary Figure 2

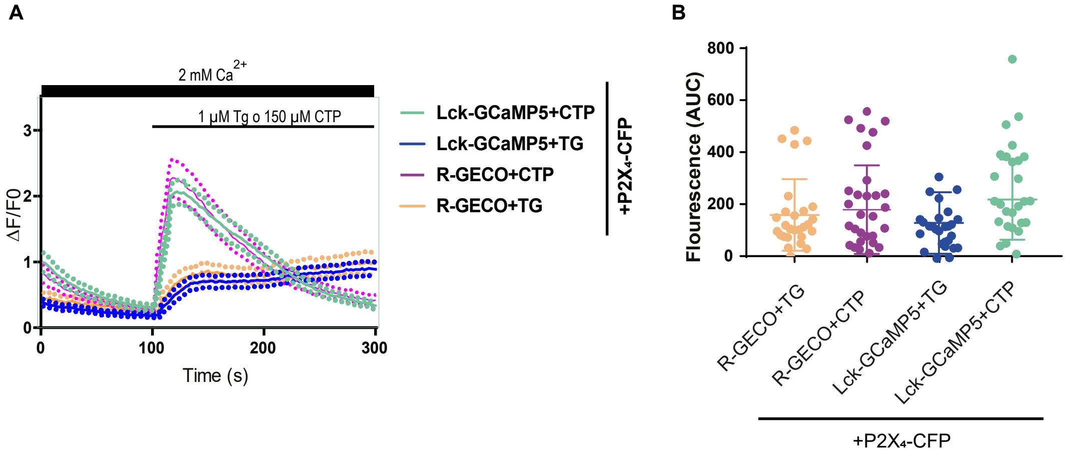

Supplementary Figure 2. The calcium sensor Lck-GCaMP5 senses CTP-induced calcium increments. A, cell populations calcium measurements using the plasma membrane targeted Lck-GCaMP5 (green) and the cytosolic R-GECO (red). Lck-GCaMP5 is a membrane-targeted GCaMP5 containing the first twenty-six amino acids of Lck, a Src family tyrosine kinase containing two palmitoylation domains and a single myristoylation domain, which target the GCaMP5 calcium sensor to the plasma membrane. **B**, area under the curve for the different conditions shown in A. In all cases data shows the mean ± standard deviation.

Supplementary Figure 3

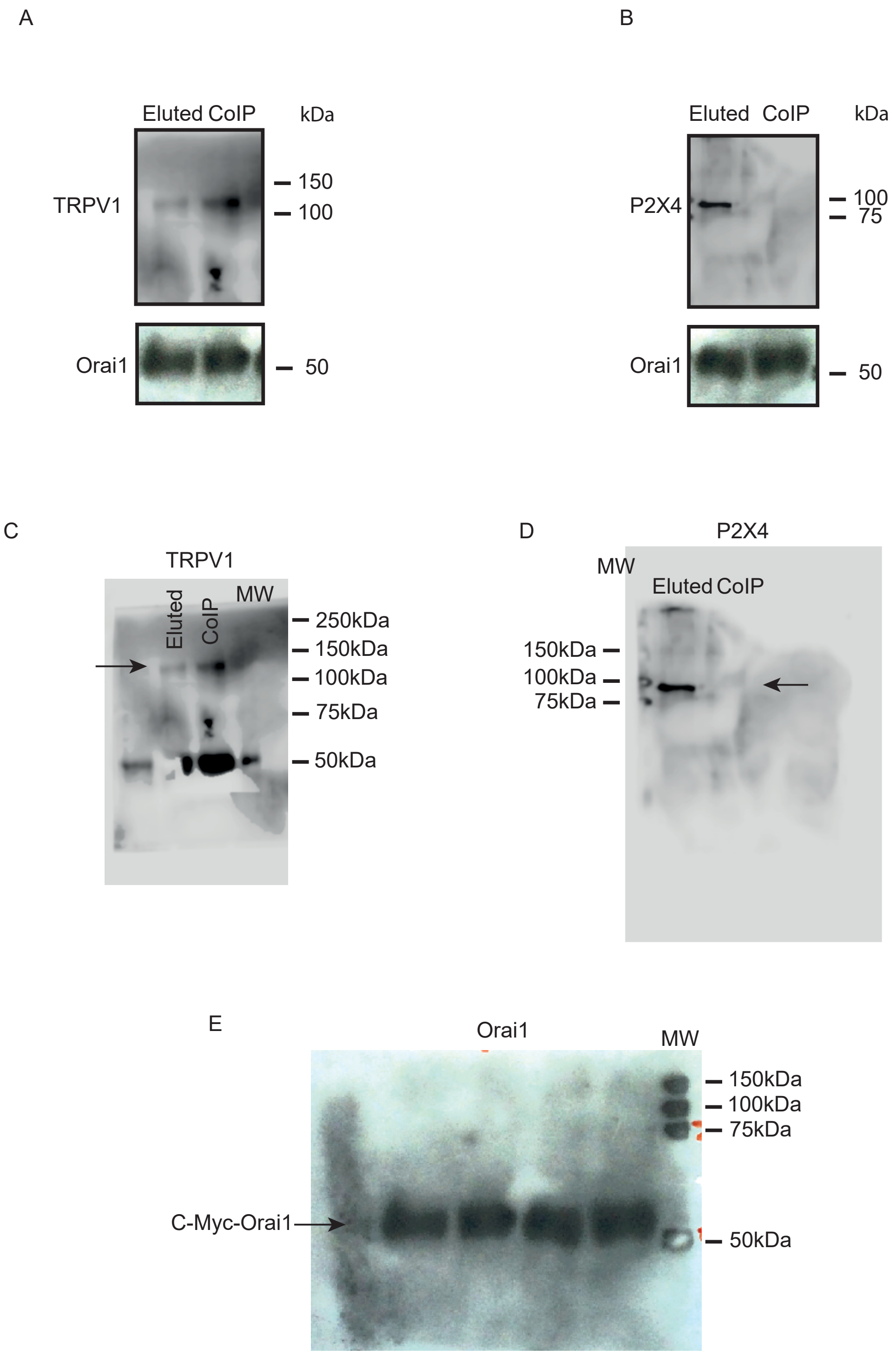

Supplementary Figure 3. Full-length western blot membranes for Orai1, TRPV1 and P2X4. **A**, western blots showing the co-immunoprecipitation of TRPV1 with c-Myc-Orai1 but not P2X4 (**B). C**, full length blots from which panel A was obtained. **D**, full length blots from which panel B was obtained. **E**, full length blots for C-Myc-Orai1 (loading control for panels A-D).

Supplementary Figure 4

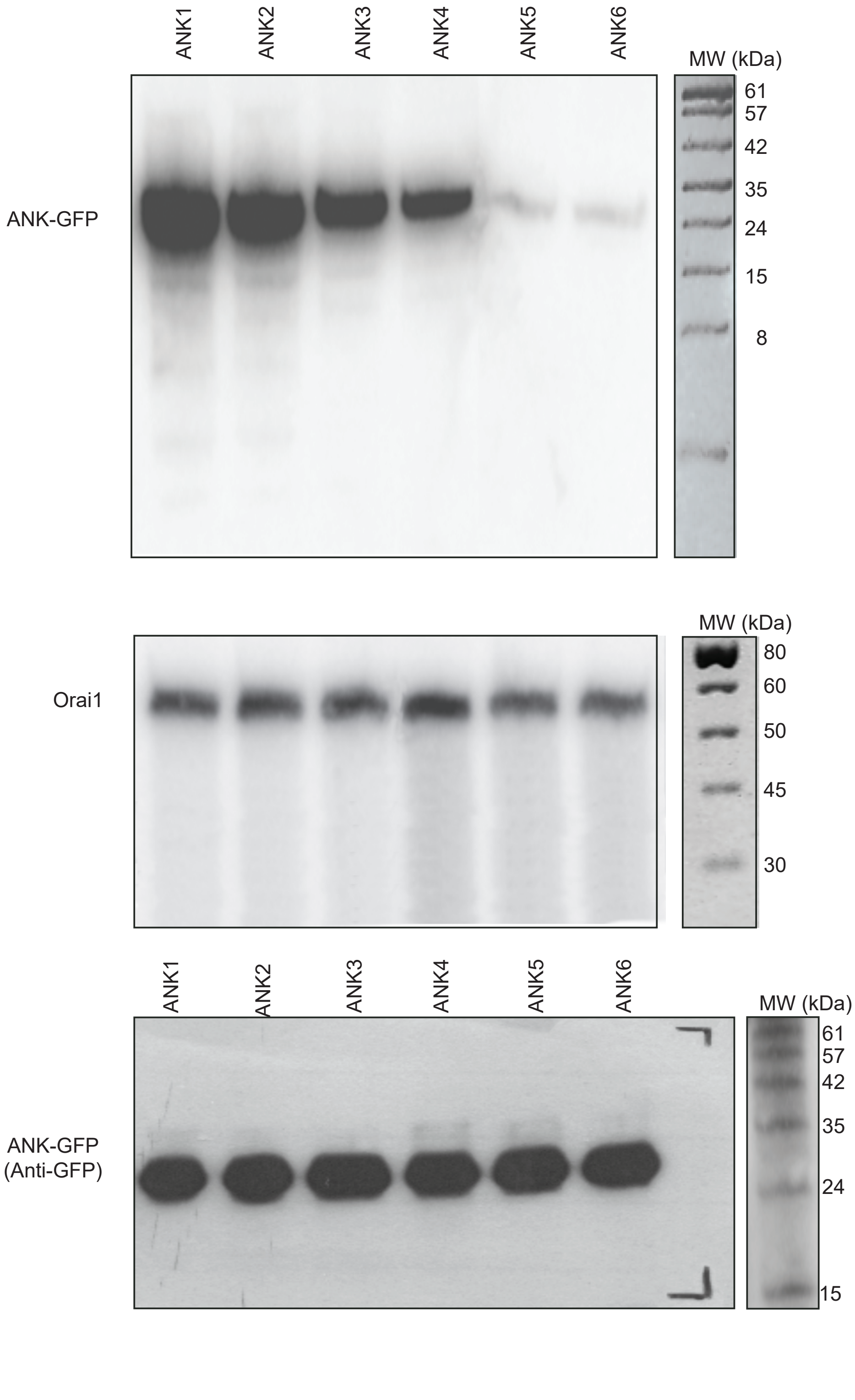

Supplementary Figure 4. **Full-length western blot membranes with ankyrin domains**. Complete western blots showing the co-immunoprecipitation of Orai1 with all 6 ankyrin domains. Lower blot shows the quantification of each ANK-GFP domain to determine that similar amounts of each ANK domain were used in the coIP studies illustrated in the upper blots. Orai1 and all ankyrin domains fused to GFP were individually transfected in HEK293 cells as described in Material and Methods.

Supplementary igure 5

HEK293 cells

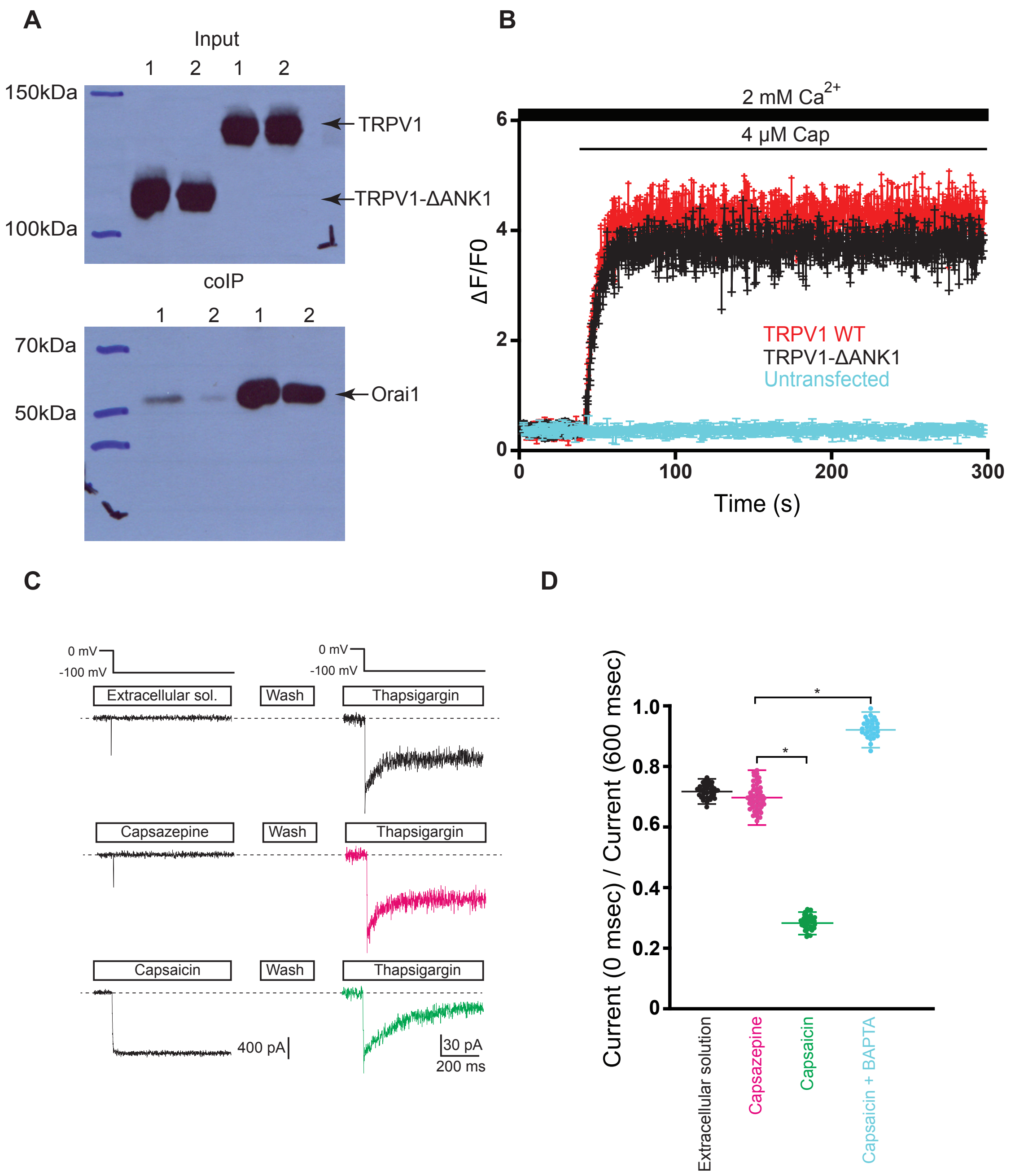

Supplementary Figure 5. Full-length western blot membranes and calcium measurements with TRPV1-ΔANK1. A, co-immunoprecipitation of Orai1 using TRPV1 wild type or TRPV1-ΔANK1. Notice that TRPV1-ΔANK1 immunoprecipitated a very small fraction of Orai1. TRPV1 wild type, TRPV1-ΔANK1 and Orai1 were transfected in HEK293 cells as described in Material and Methods. The TRPV1 (wild type) and TRPV1ΔANK1 in the input blot were detected with the specific TRPV1 antibody (sc-

398417, Material and Methods). Orai1 in the coIP blot was identified using the specific anti-Oai1 antibody (ab59330, Material and Methods). Lanes 1 and 2 show replicates from different transfection days. Blue lines are markers were the different molecular weight standards were located. **B**, calcium measurements using R-GECO in HEK293 cells transfected with TRPV1 wild type (red) or TRPV1-ΔANK1 (black). Unstransfected cells do not respond to capsaicin (green). In all cases data shows the mean ± standard deviation from at least 5 independent transfections. Calcium measurements were conducted in cell populations as described in Material and Methods. **C**, whole-cell perforated patch electrophysiology measurements in HEK293 cells expressing Orai1-GFP, STIM-dsRED and TRPV1. The sequential stimulation control protocol consisted first in the application of extracellular solution (Material and Methods) followed by a second control application of extracellular solution (WASH) and finally thapsigargin. In all cases capsaicin was incubated for 3 minutes and then the cells were washed with fresh ES for 1-2 minutes prior to TG stimulation. First top panel shows the protocol using ES as control. Middle panel shows the control using the synthetic antagonist of capsaicin, capsazepine. Lower panel shows the stimulation with capsaicin. **D**, Ratio of current measured at the beginning of the change in voltage to -100 mV (0 msec) and the current measured at 600 msec. Similar plots have been previously used to characterize CDI on Orai1 channels [46]. Data shows the mean ± standard deviation from at least 15 cells from 4 independent transfections. Asterisks show p values of <0.01. The statistical significance was $p < 0.05$ according to ANOVA.

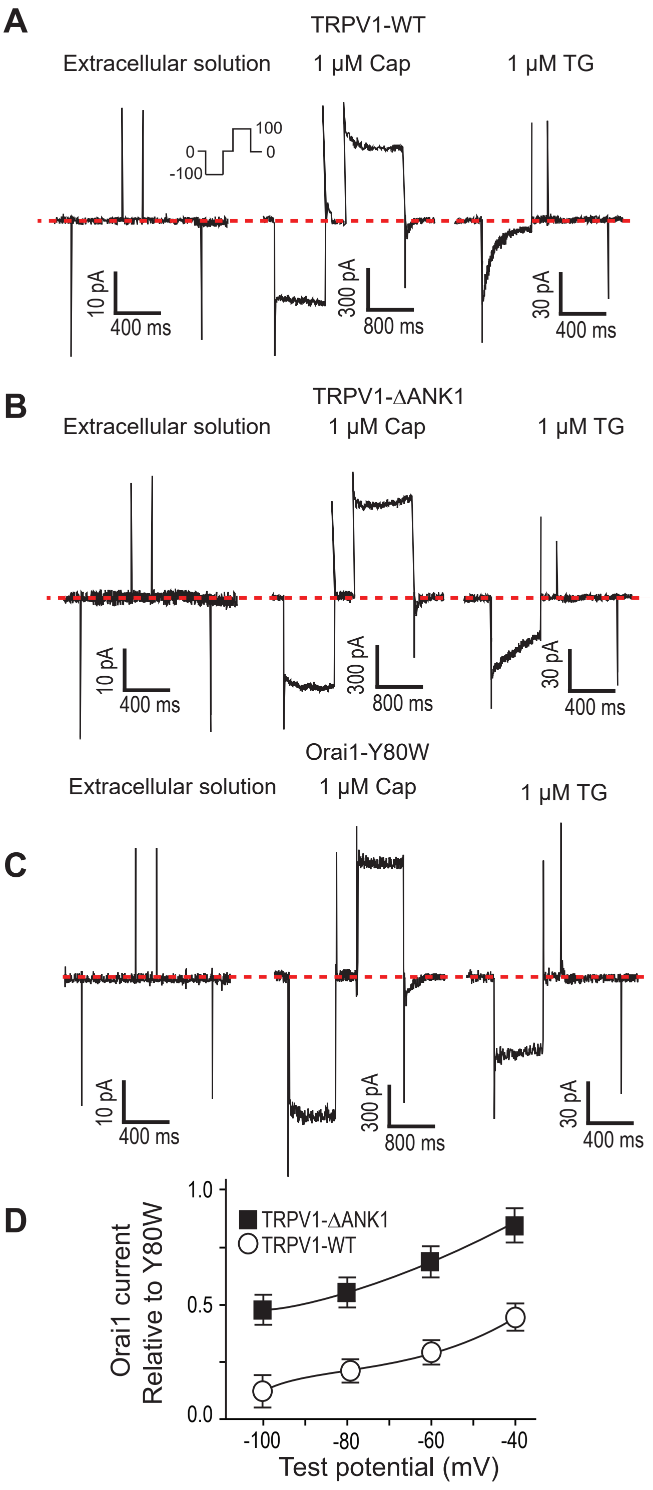

Supplementary Figure 6. TRPV1-ΔANK1 does not induce CDI in Orai1. A, perforated patch sequential protocol illustrating the induction of CDI in Orai1 when TRPV1 is stimulated prior to activating Orai1 with TG. **B**, reduced CDI in Orai1 when the deletion mutant TRPV1-ΔANK1 is activated prior to activation of Orai1 with TG. **C**, the Orai1 mutant Y80W that does not show CDI is used as control. **D**, Orai1 does not show CDI at

all voltages explored when TRPV1-ΔANK1 is expressed and activated. Data shows the mean ± standard deviation from at least 10 cells from 3 independent transfections.

Supplementary Figure 7

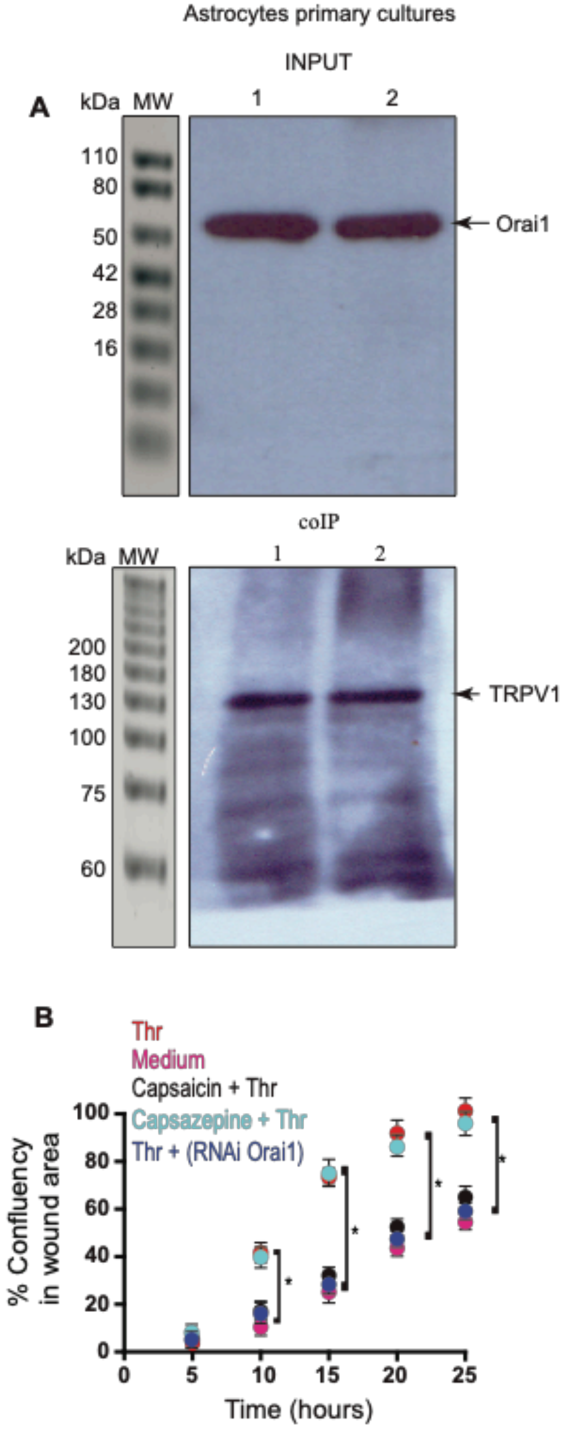

Supplementary Figure 7. Full length western blots for endogenous Orai1 and TRPV1 from cortical astrocytes. A, full-length western blots illustrating the co-immunoprecipitation of TRPV1 with Orai1 selective antibody. Lanes 1 and 2 show two independent CoIP. **B**, percentage of cell confluence measure from the scratch wound assay (SWA). Measurements of the area were obtained as described in Material and Methods from images like those illustrated in figure 7. Notice that the synthetic antagonist of capsaicin, capsazepine had no effect on thrombin-induced wound healing. Transfecting the astrocytes with a siRNAi for Orai1 (Material and Methods) prevented the thrombin-induced wound healing, indicating that this channel is required for thrombin-induced wound healing. Data shows the mean ± standard deviation from at least 6 cells from 3 independent transfections. Asterisks show p values of <0.01. The statistical significance was $p <0.05$ according to ANOVA.
