# Supplementary material for: Heterologous calcium-dependent inactivation of Orai1 by neighboring TRPV1 channels modulates cell migration and wound healing
Source: Commun Biol. 2019 Mar 4;2:88. doi: 10.1038/s42003-019-0338-1 (PMC6399350; doi:10.1038/s42003-019-0338-1)
Supplement: Supplementary file 2 — Description of Additional Supplementary Files [file 42003_2019_338_MOESM2_ESM.docx]

**Description of Additional Supplementary Files**

**File Name**: Supplementary Movie 1

**Description**: Super resolution time course experiments showing the fluorescence from Orai1-GCaMP3 (green) in cells co-expressing P2X4-CFP (purple). Each particle may represent a single channel based on the behavior and scale. Notice that activation of P2X4 with CTP does not increment the fluorescence of Orai1-GCaMP3. Compare these results with those obtained in video 2. Data from experiments like this one were used to produce figures 5 and 6.

**File Name**: Supplementary Movie 2

**Description**: Super resolution time course experiments showing the fluorescence from Orai1-GCaMP3 (green) in cells co-expressing TRPV1-CFP (blue). Each particle may represent a single channel based on the behavior and scale. Notice that activation of TRPV1 with capsaicin results in a large increment in fluorescence from Orai1-GCaMP3. Compare these results with those obtained in video 1. Data from experiments like this one were used to produce figures 5 and 6.
