# Supplementary material for: Heterologous calcium-dependent inactivation of Orai1 by neighboring TRPV1 channels modulates cell migration and wound healing
Source: Commun Biol. 2019 Mar 4;2:88. doi: 10.1038/s42003-019-0338-1 (PMC6399350; doi:10.1038/s42003-019-0338-1)
Supplement: Supplementary file 5 — Reporting Summary [file 42003_2019_338_MOESM5_ESM.pdf]

nature research

Corresponding author(s): Luis Vaca
Last updated by author(s): Feb 4, 2019

Reporting Summary

Nature Research wishes to improve the reproducibility of the work that we publish. This form provides structure for consistency and transparency in reporting. For further information on Nature Research policies, see Authors & Referees and the Editorial Policy Checklist.

Statistics

For all statistical analyses, confirm that the following items are present in the figure legend, table legend, main text, or Methods section.

n/a	Confirmed	
☐	☒	The exact sample size (n) for each experimental group/condition, given as a discrete number and unit of measurement
☐	☒	A statement on whether measurements were taken from distinct samples or whether the same sample was measured repeatedly
☐	☒	The statistical test(s) used AND whether they are one- or two-sided<br>*Only common tests should be described solely by name; describe more complex techniques in the Methods section.*
☒	☐	A description of all covariates tested
☒	☐	A description of any assumptions or corrections, such as tests of normality and adjustment for multiple comparisons
☐	☒	A full description of the statistical parameters including central tendency (e.g. means) or other basic estimates (e.g. regression coefficient) AND variation (e.g. standard deviation) or associated estimates of uncertainty (e.g. confidence intervals)
☐	☒	For null hypothesis testing, the test statistic (e.g. F, t, r) with confidence intervals, effect sizes, degrees of freedom and P value noted<br>*Give P values as exact values whenever suitable.*
☒	☐	For Bayesian analysis, information on the choice of priors and Markov chain Monte Carlo settings
☒	☐	For hierarchical and complex designs, identification of the appropriate level for tests and full reporting of outcomes
☐	☒	Estimates of effect sizes (e.g. Cohen's d, Pearson's r), indicating how they were calculated

Our web collection on statistics for biologists contains articles on many of the points above.

Software and code

Policy information about availability of computer code

Data collection	Images were collected with Fluoview or Xcellence RT from Olympus. Electrophysiology data were collected with Patch Master and EPC10 electronics software (Heka electronics).
Data analysis	images were analized with Fluoview (Olympus), Imaris, MacBiophotonics (ImageJ) or Fiji (ImageJ). Electrophysiology data were analized in Igor pro V.7 (Wavemetrics)

For manuscripts utilizing custom algorithms or software that are central to the research but not yet described in published literature, software must be made available to editors/reviewers. We strongly encourage code deposition in a community repository (e.g. GitHub). See the Nature Research guidelines for submitting code & software for further information.

Data

Policy information about availability of data

All manuscripts must include a data availability statement. This statement should provide the following information, where applicable:

- Accession codes, unique identifiers, or web links for publicly available datasets
- A list of figures that have associated raw data
- A description of any restrictions on data availability

All data supporting the findings of this study are available within the article and Supplementary Information, or from the corresponding author upon request. Aqui falta lo de los microarreglos, es decir donde estan.

Field-specific reporting

Please select the one below that is the best fit for your research. If you are not sure, read the appropriate sections before making your selection.

☒ Life sciences ☐ Behavioural & social sciences ☐ Ecological, evolutionary & environmental sciences

For a reference copy of the document with all sections, see nature.com/documents/nr-reporting-summary-flat.pdf

Life sciences study design

All studies must disclose on these points even when the disclosure is negative.

Sample size	Sample-size calculations were not required in the case of this study. Experiments were independently repeated at least three times as indicated, and mean and the standard deviation from the mean were calculated.
Data exclusions	Experiments were excluded that were technically invalid.
Replication	The replication of experiments was successful.
Randomization	Randomization was not relevant for this study.
Blinding	Blinding was not relevant for this study.

Reporting for specific materials, systems and methods

We require information from authors about some types of materials, experimental systems and methods used in many studies. Here, indicate whether each material, system or method listed is relevant to your study. If you are not sure if a list item applies to your research, read the appropriate section before selecting a response.

Materials & experimental systems

n/a	Involved in the study	
☐	☒	Antibodies
☐	☒	Eukaryotic cell lines
☒	☐	Palaeontology
☐	☒	Animals and other organisms
☒	☐	Human research participants
☒	☐	Clinical data

Methods

n/a	Involved in the study	
☒	☐	ChIP-seq
☒	☐	Flow cytometry
☒	☐	MRI-based neuroimaging

Antibodies

Antibodies used	Orai1 (ab59330, Abcam), c-myc (Invitrogen, MA1-21316), VR1 (Santa Cruz, 398417) and P2X4 (Abcam, 134559) for CFP-P2X4 (71 kDa)
Validation	Validation was conducted using anti-Myc and anti-GFP antibodies to corroborate recognition of the same molecular weight band as that recognized by the selective antibodies anti-Orai1 and anti-TRPV1

Eukaryotic cell lines

Policy information about cell lines

Cell line source(s)	ATCC
Authentication	None of the cell lines used were authenticated
Mycoplasma contamination	Cell lines were tested for mycoplasma contamination
Commonly misidentified lines (See ICLAC register)	None of the misidentified cell lines were used in this study

Animals and other organisms

Policy information about studies involving animals; ARRIVE guidelines recommended for reporting animal research

Laboratory animals	Neonatal male rats (4- 8 days)
Wild animals	No wild animals were used for this study.
Field-collected samples	This study did not involve samples collected from field.
Ethics oversight	Animal Care Committee of the Instituto de Fisiología Celular, Universidad Nacional Autónoma de México

Note that full information on the approval of the study protocol must also be provided in the manuscript.
